# Monocyte metabolic reprogramming promotes pro-inflammatory activity and *Staphylococcus aureus* biofilm clearance

**DOI:** 10.1371/journal.ppat.1008354

**Published:** 2020-03-06

**Authors:** Kelsey J. Yamada, Cortney E. Heim, Xinyuan Xi, Kuldeep S. Attri, Dezhen Wang, Wenting Zhang, Pankaj K. Singh, Tatiana K. Bronich, Tammy Kielian

**Affiliations:** 1 Department of Pathology and Microbiology, University of Nebraska Medical Center, Omaha, Nebraska, United States of America; 2 Department of Pharmaceutical Sciences, University of Nebraska Medical Center, Omaha, Nebraska, United States of America; 3 Eppley Institute, University of Nebraska Medical Center, Omaha, Nebraska, United States of America; University of Tubingen, GERMANY

## Abstract

Biofilm-associated prosthetic joint infections (PJIs) cause significant morbidity due to their recalcitrance to immune-mediated clearance and antibiotics, with *Staphylococcus aureus* (*S*. *aureus*) among the most prevalent pathogens. We previously demonstrated that *S*. *aureus* biofilm-associated monocytes are polarized to an anti-inflammatory phenotype and the adoptive transfer of pro-inflammatory macrophages attenuated biofilm burden, highlighting the critical role of monocyte/macrophage inflammatory status in dictating biofilm persistence. The inflammatory properties of leukocytes are linked to their metabolic state, and here we demonstrate that biofilm-associated monocytes exhibit a metabolic bias favoring oxidative phosphorylation (OxPhos) and less aerobic glycolysis to facilitate their anti-inflammatory activity and biofilm persistence. To shift monocyte metabolism *in vivo* and reprogram cells to a pro-inflammatory state, a nanoparticle approach was utilized to deliver the OxPhos inhibitor oligomycin to monocytes. Using a mouse model of *S*. *aureus* PJI, oligomycin nanoparticles were preferentially internalized by monocytes, which significantly reduced *S*. *aureus* biofilm burden by altering metabolism and promoting the pro-inflammatory properties of infiltrating monocytes as revealed by metabolomics and RT-qPCR, respectively. Injection of oligomycin alone had no effect on monocyte metabolism or biofilm burden, establishing that intracellular delivery of oligomycin is required to reprogram monocyte metabolic activity and that oligomycin lacks antibacterial activity against *S*. *aureus* biofilms. Remarkably, monocyte metabolic reprogramming with oligomycin nanoparticles was effective at clearing established biofilms in combination with systemic antibiotics. These findings suggest that metabolic reprogramming of biofilm-associated monocytes may represent a novel therapeutic approach for PJI.

## Author summary

Prosthetic joint infections (PJIs) are a catastrophic complication following joint replacement surgery and are predominantly caused by staphylococcal species, such as *S*. *aureus*. The morbidity associated with PJI results, in large part, from the inability to clear infection, as bacteria are organized into heterogeneous communities known as biofilms, which are recalcitrant to antibiotic therapy and immune-mediated clearance. This study revealed that biofilm-associated monocytes exhibit a metabolic bias towards oxidative phosphorylation (OxPhos), which is linked to their anti-inflammatory properties. We developed a nanoparticle delivery approach using the OxPhos inhibitor oligomycin, which significantly reduced biofilm burden by promoting the pro-inflammatory properties of infiltrating monocytes in a mouse model of *S*. *aureus* PJI. Notably, nanoparticle delivery combined with systemic antibiotics reduced biofilm titers in mice to below the limit of detection. These findings suggest that leukocyte metabolic reprogramming could represent a novel therapeutic avenue for circumventing the two-stage revision protocol for patients with PJI by treating an infected implant *in situ* and alleviating a second surgery, which would represent a significant reduction in patient morbidity.

## Introduction

The frequency of orthopedic procedures, such as total knee and total hip arthroplasty (TKA and THA, respectively), continues to increase and is predicted to reach an annual rate in the United States by 2030 of 572,000 and 3.48 million, respectively [1]. Although most surgeries are successful, devices may fail following prosthetic joint infection (PJI), a complication that can cause sustained disability and increased health care costs attributable to prolonged antibiotic treatment and multiple surgeries. The estimated annual incidence of PJI in the United States is 2.18% for all THAs and TKAs [2]; however, the infection rate following revision surgery is even higher (i.e. 3.2–5.6% for both THA and TKAs). The current standard-of-care for PJIs includes removal of the infected hardware, placement of an antibiotic-impregnated polymethylmethacrylate spacer for 4–6 weeks until the infection has resolved, followed by a second surgery to insert a new prosthesis [3–5]. This demonstrates the importance of developing novel therapeutic approaches to treat biofilm-associated infections without requiring multiple surgical interventions.

*S*. *aureus* is a leading cause of PJI that is characterized by biofilm formation [5], and with the increased prevalence of methicillin-resistant *S*. *aureus* (MRSA) this pathogen has become an even greater therapeutic challenge [6]. Biofilm infections persist despite antibiotic therapy due, in part, to decreased metabolic activity of a subpopulation of biofilm-associated bacteria and evasion of the immune response [5,7–10]. Our laboratory has demonstrated that *S*. *aureus* biofilms skew the host immune response toward an anti-inflammatory state [11]. This is evident by the polarization of anti-inflammatory monocytes/macrophages (MΦs) and significant recruitment of myeloid-derived suppressor cells (MDSCs) [12–15]. Two major MDSC subsets have been described, namely granulocytic and monocytic MDSCs (G-MDSCs and M-MDSCs, respectively) that possess the capacity to differentiate into mature granulocytes or MΦs, respectively, given the appropriate environmental cues [16,17]. However, during chronic pathological conditions, such as cancer, infection, or autoimmune disorders, MDSCs are often arrested in an immature state where they can inhibit T cell activation and monocyte/MΦ pro-inflammatory properties [18–20]. We have demonstrated that G-MDSCs are integral to *S*. *aureus* biofilm establishment and persistence and polarize infiltrating monocytes towards an anti-inflammatory phenotype [12–15]. These effects are mediated, in part, via IL-10 production by MDSCs, which can attenuate NF-κB activation in monocytes and MФs and down-regulate cytokine expression [12]. In addition, reductions in MDSC infiltrates augment monocyte pro-inflammatory activity, which translates into reduced biofilm burden [12–14]. Biofilm clearance is also facilitated by the adoptive transfer of pro-inflammatory MΦs at the site of infection [21]. Therefore, the immune polarization state of monocytes and MΦs plays a key role in dictating biofilm persistence and this is influenced, in part, by MDSC-derived factors. Our prior work has identified the presence of monocyte, MDSC, and neutrophil (PMN) infiltrates in human PJI, revealing similarities between the mouse model and human infection [22].

Recent studies have shown that the inflammatory phenotype of MΦs is intimately tied to their metabolic state. For example, anti-inflammatory MΦs rely primarily on oxidative phosphorylation (OxPhos) to drive their suppressive activity [23,24]. However, upon exposure to pro-inflammatory stimuli, MΦs favor aerobic glycolysis. Of note, monocytes outnumber MΦs in our mouse PJI model and we have shown that monocytes are also polarized toward an anti-inflammatory state during *S*. *aureus* biofilm infection [12–15]. However, it is not known whether these anti-inflammatory monocytes can be metabolically reprogrammed to promote their pro-inflammatory properties and biofilm clearance. Monocyte metabolism and inflammatory phenotypes exist within a spectrum and adapt in response to environmental stimuli [25,26]. Here we show that monocytes infiltrating *S*. *aureus* biofilms exhibit a metabolic bias towards OxPhos, with a concomitant decrease in glycolysis. To demonstrate that the metabolic profile of biofilm-associated monocytes can be modified to promote their pro-inflammatory activity, we utilized nanoparticles containing the OxPhos inhibitor, oligomycin [27]. To target nanoparticle uptake to biofilm-associated monocytes *in vivo*, nanoparticles were conjugated with tuftsin, a tetrapeptide derived from the Fc domain of the IgG heavy chain [28], and also incorporated a fluorochrome (Cy5) to monitor nanoparticle distribution. *In vivo*, Cy5-labeled oligomycin nanoparticles were preferentially internalized by monocytes, with minimal uptake by MDSCs or PMNs. Using a mouse model of *S*. *aureus* PJI, oligomycin nanoparticles inhibited monocyte OxPhos activity and shifted cells to a pro-inflammatory state concomitant with increased PMN and monocyte recruitment, resulting in a significant reduction in *S*. *aureus* burden. These effects were not recapitulated with free oligomycin, establishing that intracellular delivery of oligomycin to monocytes is required and that oligomycin does not exert any direct bactericidal activity against *S*. *aureus*. The combined treatment of mice with oligomycin nanoparticles and systemic antibiotics cleared an established biofilm infection, a finding that to the best of our knowledge has never been achieved for a mature *S*. *aureus* biofilm *in vivo*. Collectively, monocyte metabolic reprogramming could represent a novel therapeutic approach for circumventing the two-stage revision protocol for patients with PJI by treating an infected implant *in situ* and alleviating a second surgery, which would represent a significant reduction in patient morbidity.

## Results

### *S*. *aureus* biofilm infection promotes a shift towards OxPhos metabolism in monocytes

**Fig 1:**
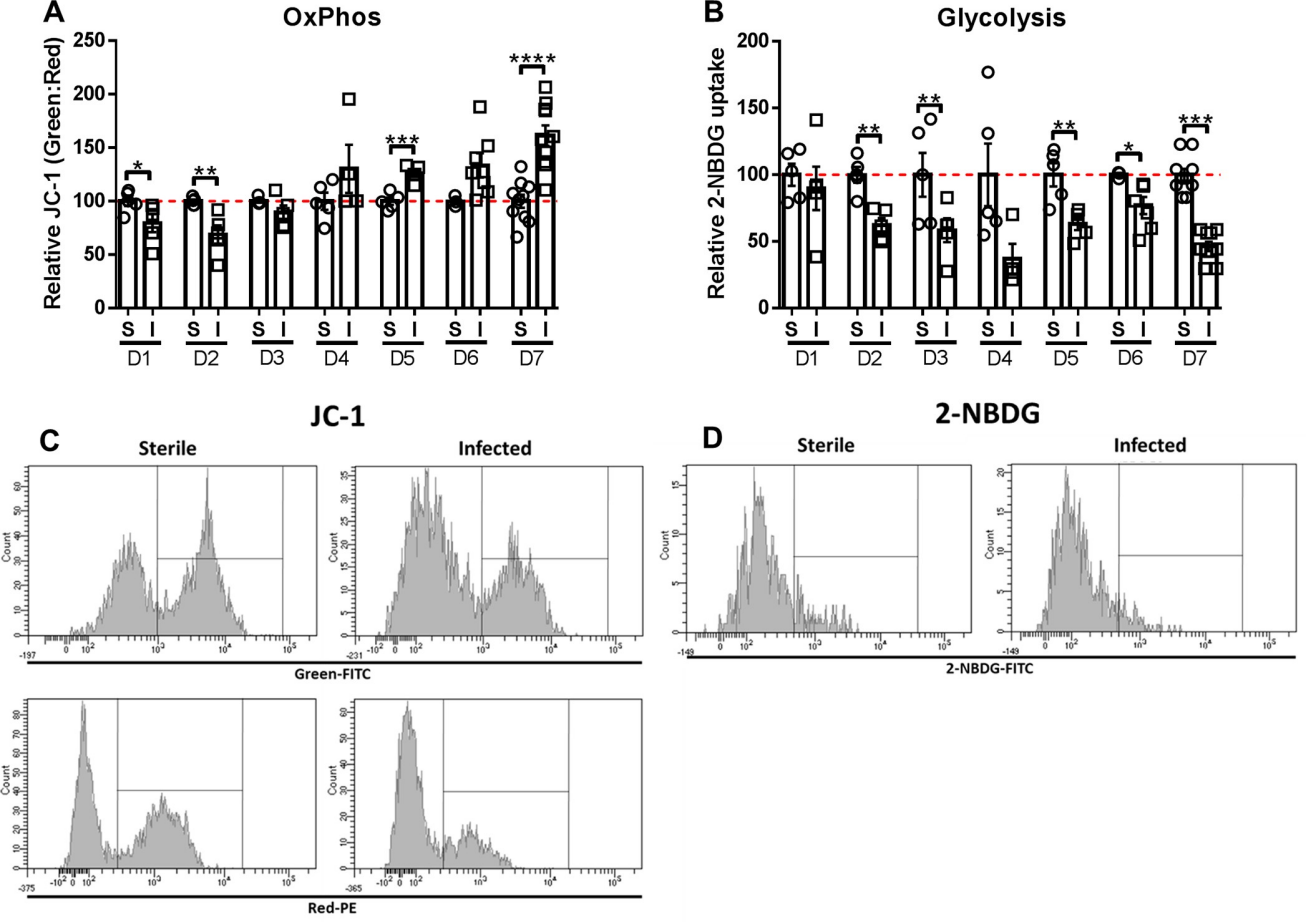
*S*. *aureus* biofilm infection promotes a shift towards OxPhos metabolism in monocytes. Monocytes associated with tissues surrounding the knee joint of mice with sterile (S) or *S*. *aureus-*infected (I) orthopedic implants were stained with (**A**) the bi-potential dye JC-1 or (**B**) 2-NBDG at the indicated time points (days 1–7) as measures of OxPhos and glycolysis, respectively, and analyzed by flow cytometry. (**A**) OxPhos was calculated as the ratio of green:red monocytes (CD11b^high^Ly6G^-^Ly6C^+^F4/80^-^) following JC-1 staining and is reported as a percentage relative to animals receiving sterile implants. (**B**) Glycolytic activity is reported as the percentage of monocytes that were 2-NBDG^+^ relative to mice with sterile implants. Representative histograms of (**C**) JC-1 and (**D**) 2-NBDG at day 7 are shown. Data are presented as the mean ± SD (n = 3–10 animals/group) combined from two independent experiments (*, *p* < 0.05; **, *p* < 0.01; ***, *p* < 0.001; ****, *p* < 0.0001; Student’s *t*-test).

Previous work from our laboratory using a mouse model of *S*. *aureus* PJI has shown that biofilms promote monocyte anti-inflammatory activity [12–14]. To determine whether this anti-inflammatory bias is associated with a metabolic shift favoring OxPhos over glycolysis, the metabolic profiles of monocytes associated with *S*. *aureus* infected vs. sterile orthopedic implants were examined. The rationale for this comparison was two-fold; first, the placement of a sterile implant elicits a pro-inflammatory tissue injury response [13], which could be compared with the anti-inflammatory biofilm milieu. Second, evaluating the metabolic state of monocytes isolated from *S*. *aureus* biofilms versus abscesses would lead to confounds because of differences in bacterial burdens between the models and the fact that abscesses are cleared, whereas biofilm infections remain chronic. Seahorse metabolic assays are often used to evaluate cellular metabolic states; however, this approach was not feasible given the limited numbers of implant-associated monocytes *in vivo*. Therefore, flow cytometry was performed with JC-1 and 2-NBDG, validated tools that are used in the immunometabolism field as indicators of cellular OxPhos and glycolytic activity, respectively [29,30]. JC-1 is a bi-potential dye that accumulates in mitochondria with large membrane potentials and fluoresces red, whereas depolarized mitochondria are green. Therefore, cells exhibiting increased OxPhos activity display a larger green:red ratio [31]. OxPhos was significantly increased in monocytes infiltrating *S*. *aureus* biofilms compared to sterile implants at later time points (i.e. days 5–7; Fig 1A and 1C), which our prior studies have demonstrated coincides with biofilm maturation as measured by antibiotic tolerance [15]. Interestingly, monocyte OxPhos activity was significantly reduced during the first two days of infection (Fig 1A), which may reflect an initial pro-inflammatory response that transforms over time to an anti-inflammatory state. Glycolysis was evaluated by the uptake of the fluorescent glucose analog 2-NBDG [32], which revealed significant and persistent reductions in glycolytic activity in *S*. *aureus* biofilm-associated monocytes compared to sterile implants (Fig 1B and 1D). Collectively, increased OxPhos concomitant with reduced glycolysis in biofilm-associated monocytes corresponds with their reported anti-inflammatory properties [12–14].

#### Inhibition of OxPhos by oligomycin promotes MФ pro-inflammatory activity

**Fig 2:**
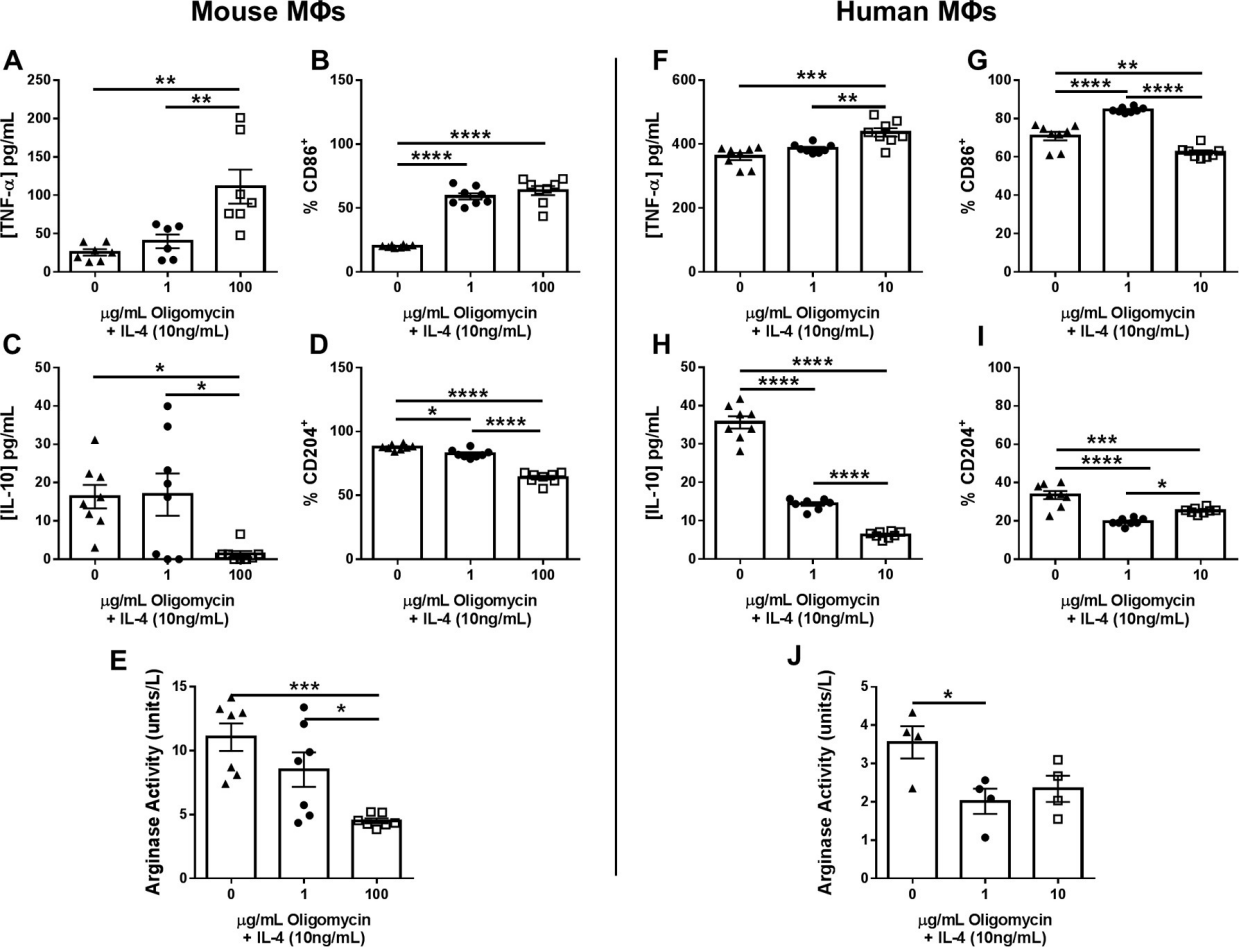
Inhibition of OxPhos by oligomycin promotes MФ pro-inflammatory activity. Mouse bone marrow-derived or human monocyte-derived MФs were plated overnight and the following day cells were pre-treated with IL-4 (10 ng/mL) for 1 h followed by various concentrations of oligomycin for 24 h, whereupon TNF-α (A and F) and IL-10 (C and H) production was determined by cytometric bead array or ELISA, CD86 (B and G) and CD204 (D and I) expression by flow cytometry, and arginase activity by an enzymatic assay (E and J). Data are presented as the mean ± SD combined from two independent experiments (n = 4–8 biological replicates) (*, *p* < 0.05; **, *p* < 0.01; ***, *p* < 0.001; ****, *p* < 0.0001; Student’s *t*-test).

Since biofilm-associated monocytes displayed an OxPhos bias, we explored whether this could be attenuated to promote their pro-inflammatory activity. Oligomycin inactivates mitochondrial ATP synthase of the electron transport chain to inhibit OxPhos [27,33]. Prior work has demonstrated that oligomycin shifts MФs towards aerobic glycolysis, which coincides with pro-inflammatory gene expression [27]. To establish the efficacy of oligomycin in reprogramming MФ inflammatory properties, mouse bone marrow-derived and human monocyte-derived MФs were treated with the anti-inflammatory cytokine IL-4 in the presence/absence of oligomycin. Oligomycin reversed the anti-inflammatory effects of IL-4 in a dose-dependent manner in both mouse and human MФs as evident by significant increases in TNF-α and CD86 (Fig 2A, 2B, 2F and 2G) concomitant with decreased IL-10 and CD204 expression (Fig 2C, 2D, 2H and 2I) and arginase activity (Fig 2E and 2J), hallmark molecules of pro- and anti-inflammatory MФs, respectively [34]. In addition, gentamicin protection assays revealed that oligomycin treatment enhanced the anti-bacterial activity of both human and mouse MФs (S1 Fig).

#### Tuftsin-conjugated nanoparticles are preferentially internalized by monocytes during *S. aureus* PJI

Given the ability of oligomycin to promote MФ pro-inflammatory activity, we designed a nanoparticle delivery approach using tuftsin as a targeting moiety, to deliver oligomycin to biofilm-associated monocytes/MФs *in vivo* to enhance their pro-inflammatory activity and promote biofilm clearance. Tuftsin is a peptide derived from the Fc portion of IgG, which has been shown to facilitate nanoparticle internalization by MФs through its interaction with Fc receptors [28]. Previous studies have utilized tuftsin-conjugated nanoparticles to deliver a variety of biomolecules to monocytes and MФs in the context of arthritis, HIV, and other inflammatory diseases [28,35–37]. We utilized three nanoparticle types, namely Cy5 (C), Cy5/Tuftsin (CT), and Cy5/Tuftsin/Oligomycin (CTO), where the former two represented control formulations and all three harbored the Cy5 fluorochrome to identify nanoparticle localization *in vivo* (Table 1).

**Table 1:** Nanoparticle features.

Abbreviation	Composition	Description
C	Cy5	fluorochrome
CT	Cy5/Tuftsin	fluorochrome + targeting
CTO	Cy5/Tuftsin/Oligomycin	fluorochrome + targeting + ETC inhibitor

**Fig 3:**
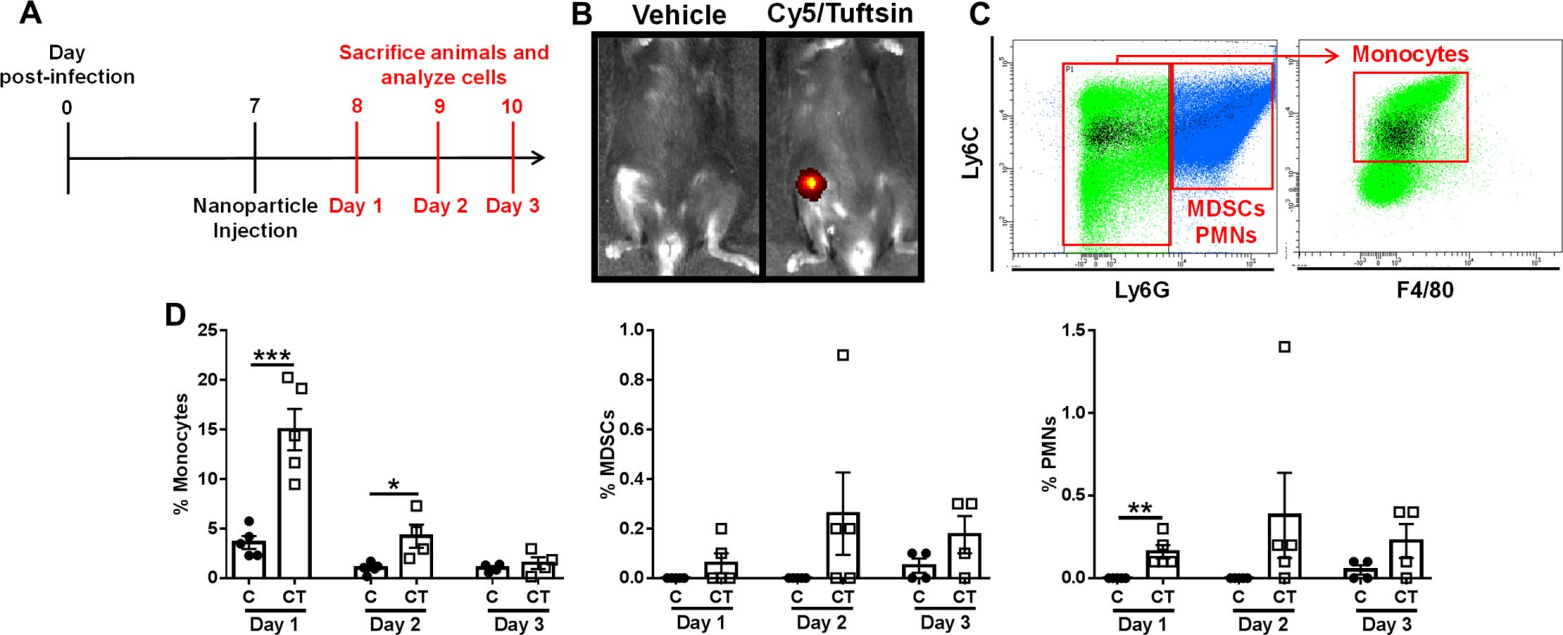
Preferential nanoparticle uptake by monocytes during PJI. (**A**) C57BL/6NCrl mice received a single intra-articular injection of Cy5 (C) or Cy5/Tuftsin (CT) nanoparticles (10 μg) at day 7 post-infection and were analyzed on three consecutive days. (**B**) IVIS imaging of CT labeled nanoparticles in the infected joint at day 3 with (**C**) representative flow cytometry dot plots. Left panel shows all CD45^+^ cells and the right panel depicts Ly6G^-^Ly6C^+^ gated cells, where Cy5^+^ monocytes (CD11b^high^Ly6G^-^Ly6C^+^F4/80^-^) are highlighted in black. (**D**) Mice receiving C or CT nanoparticles were sacrificed at days 1–3 following nanoparticle injection, whereupon Cy5^+^ monocytes, MDSCs, and PMNs were quantified by flow cytometry. Data is presented as the mean ± SD (n = 4–5 mice/time point). (*, *p* < 0.05; **, *p* < 0.01; ***, *p* < 0.001; Student’s *t*-test).

As previously mentioned, monocytes are significantly more abundant in the mouse PJI model compared to MФs (approximately 10% versus < 1% of the total CD45^+^ infiltrate, respectively)[12–15]; therefore, to determine whether tuftsin-conjugated nanoparticles are targeted to monocytes *in vivo* during *S*. *aureus* PJI, mice received a single intra-articular injection of C or CT nanoparticles at day 7 post-infection, a time point corresponding to biofilm establishment [15], and were sacrificed 1–3 days later to assess nanoparticle uptake (Fig 3A). Local nanoparticle delivery was selected over systemic administration to avoid altering monocyte/MФ activity in non-infected tissues. IVIS imaging demonstrated nanoparticle retention in the joint at day 3 post-injection (Fig 3B) and flow cytometry revealed that the majority of Cy5^+^ cells were monocytes (CD11b^high^Ly6G^-^Ly6C^+^F4/80^-^; Fig 3C). Importantly, uptake of tuftsin-conjugated (CT) nanoparticles by biofilm-associated monocytes was significantly higher compared to non-coated (C) nanoparticles, where approximately 15% of biofilm-associated monocytes were Cy5^+^ at 24 h after CT nanoparticle injection, which steadily declined to < 3.5% by 72 h (Fig 3D). The declination in intracellular Cy5 signal over time likely results from fluorescence quenching in the phagosome, which has been reported in other studies [38]; however, IVIS detected Cy5^+^ nanoparticles out to day 12 post-injection (S2B Fig), suggesting that they may act as a depot for continued action. The preferential uptake of tuftsin-modified nanoparticles by monocytes was approximately 37.5-fold higher compared to PMNs or MDSCs (< 0.4%; Fig 3D). As was observed with monocytes, the tuftsin modification (CT) did enhance nanoparticle internalization in the minor population of MDSCs and PMNs that were Cy5^+^ compared to non-conjugated nanoparticles (C). Bacterial burden during these acute intervals post-infection when nanoparticle uptake was assessed revealed a significant expansion of *S*. *aureus* within a 24 h period from the initial 1,000 cfu inoculum (~ 10^8^ cfu in implant-associated tissue), whereupon titers reached a plateau (S3A Fig). To assess which cell types were phagocytic *in vivo*, mice were infected with a *S*. *aureus* USA300 LAC-dsRed strain to quantify phagocytosis at day 3 post-infection. The majority of dsRed^+^ leukocytes were monocytes and MDSCs, although phagocytic cells represented a minority of each population (S3B Fig). Although PMNs displayed minimal nanoparticle uptake, this did not translate into altered function, since CTO nanoparticles did not augment their antibacterial activity (S1 Fig). In contrast, free oligomycin enhanced *S*. *aureus* killing by PMNs; however, the effects were less potent compared to MФs (1-log fewer bacteria in oligomycin-treated MФs vs. PMNs at 6 h; S1 Fig) and no significant alterations in TNF-α, IL-10, or arginase activity were observed in PMNs following oligomycin treatment (S4 Fig). Collectively, these data demonstrate that tuftsin conjugation confers specificity in targeting nanoparticles to monocytes and augments nanoparticle uptake to promote their pro-inflammatory and bactericidal activity.

#### Oligomycin-containing nanoparticles re-program biofilm-associated monocytes towards a pro-inflammatory phenotype *in vivo*

**Fig 4:**
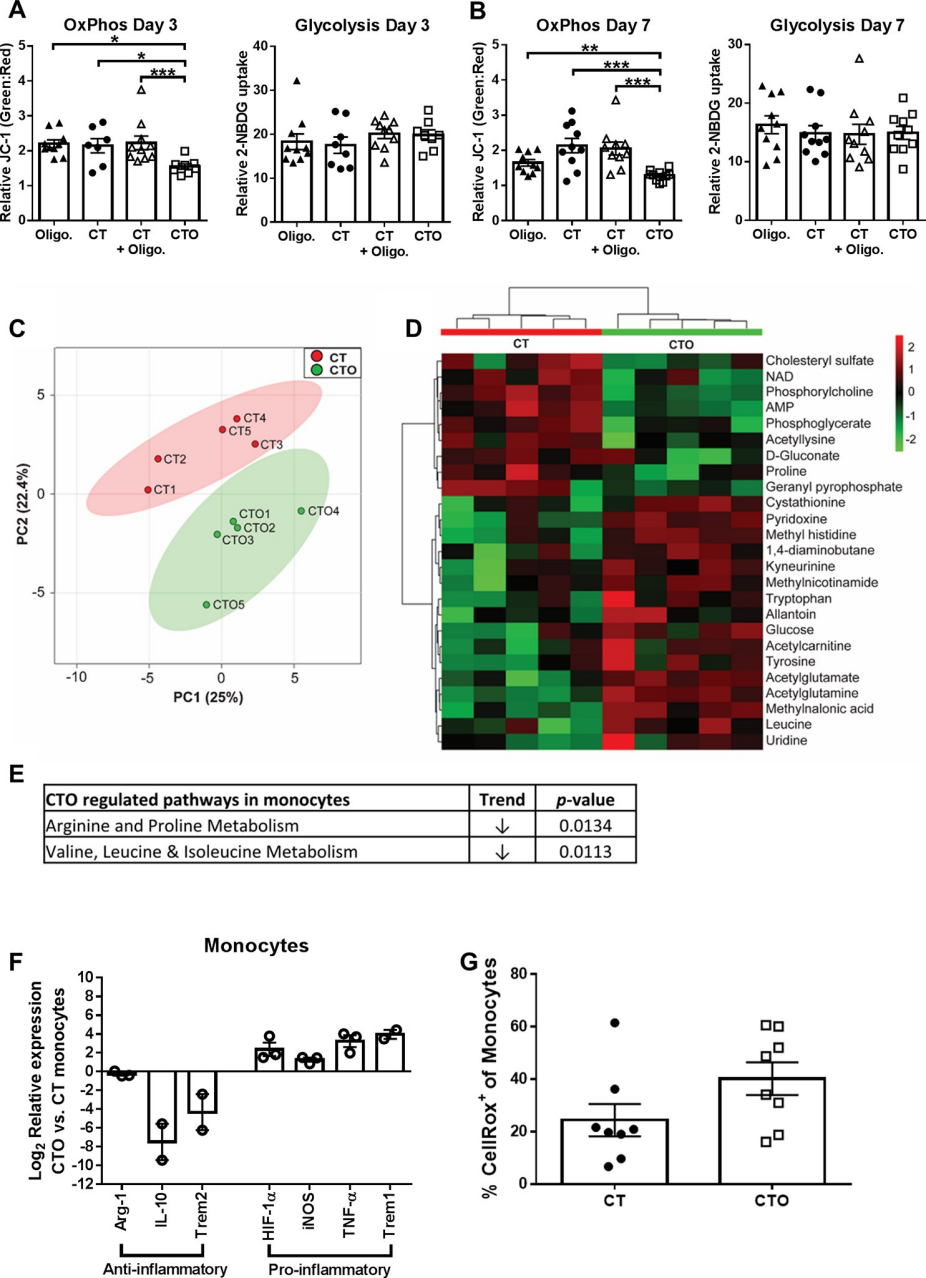
Oligomycin-containing nanoparticles induce a metabolic shift in biofilm-associated monocytes that polarizes cells towards a pro-inflammatory phenotype *in vivo*. (**A-B**) Nanoparticle-mediated delivery of oligomycin attenuates OxPhos metabolism in biofilm-associated monocytes. C57BL/6NCrl mice received a single intra-articular injection of free oligomycin only (Oligo; 100 ng), empty nanoparticles (CT), empty nanoparticles (CT) + free oligomycin (100 ng; not loaded), or oligomycin loaded nanoparticles (CTO) at day 7 post-infection and were analyzed at day 3 (**A**; 7–10 mice/group) or day 7 (**B**; 10 mice/group) after treatment. OxPhos was calculated as the ratio of green:red monocytes (CD11b^high^Ly6G^-^Ly6C^+^F4/80^-^) following JC-1 staining and glycolytic activity was calculated as the percentage of 2-NBDG^+^ monocytes. (**C-G**) C57BL/6NCrl mice received a single intra-articular injection of CT or CTO nanoparticles (10 μg) at day 7 post-infection. Mice were sacrificed 3 days following nanoparticle injection and monocytes (CD11b^high^Ly6G^-^Ly6C^+^F4/80^-^) were recovered from each individual animal by FACS (n = 5 mice/treatment group), whereupon intracellular metabolites were isolated for analysis by HPLC-MS/MS. (**C**) A principle component analysis (PCA) plot was generated using an algorithm in MetaboAnalyst with mean intensities and pareto scaling distribution. Ellipses represent a 95% confidence interval of the normal distribution for each cluster. (**D**) The heat map depicts the top 25 metabolite differences in monocytes recovered from CTO and CT treated mice. The color key indicates log_2_-fold changes of normalized mean peak intensities for metabolites in monocytes from CTO vs. CT nanoparticle treated animals. (**E**) Identification of the most significantly altered pathways in monocytes isolated from CTO treated mice as determined by pathway impact analysis. (**F**) Gene expression levels in monocytes pooled from 5 CTO-treated animals were calculated after normalizing signals to GAPDH and are presented as the fold-change relative to monocytes isolated from mice receiving CT (control) nanoparticles. Data is presented as the mean ± SD of monocytes combined from three independent experiments (n = 2–3, since some genes did not amplify in one experiment). (**G**) Reactive oxygen species production by monocytes was examined using CellROX Green (n = 8 mice).

To determine whether the selective uptake of tuftsin-conjugated oligomycin (CTO) nanoparticles by monocytes *in vivo* translated into metabolic reprogramming, metabolic assessments were performed. For these studies, mice received a single intra-articular injection of CT or CTO nanoparticles at day 7 post-infection and were sacrificed 3 days later, whereupon OxPhos and glycolytic activity were assessed by JC-1 and 2-NBDG staining, respectively. This interval was selected because our earlier studies determined that CTO nanoparticles had no impact on bacterial burden at day 3 post-injection (S5 Fig), ensuring that any differences in monocyte activation reflected effects of CTO nanoparticles rather than alterations in bacterial abundance, which was significantly altered by day 7 (S5 Fig). All biofilm-associated monocytes were analyzed, since Cy5^+^ monocytes were less frequent at 3 days following nanoparticle injection (Fig 3D), which precluded FACS enrichment. Mice were also treated with free oligomycin or empty nanoparticles (CT) with free oligomycin to confirm that intracellular delivery of oligomycin was required to modulate monocyte metabolism. Only CTO nanoparticles led to significant reductions in monocyte OxPhos activity, as revealed by JC-1 staining (Fig 4A and 4B). Overall, no changes in glycolytic activity were detected with CTO nanoparticles (Fig 4A and 4B); however, the reductions in OxPhos support a net glycolytic bias in monocytes following CTO treatment. These findings confirmed that CTO nanoparticles were effective at attenuating monocyte OxPhos *in vivo*, which was dependent on intracellular delivery of oligomycin.

Next, polar metabolites were isolated from FACS-purified monocytes (CD11b^high^Ly6C^+^Ly6G^-^ F4/80^-^) recovered from mice receiving control (CT) or CTO nanoparticles and analyzed by LC-MS/MS. Principle component analysis (PCA) using unsupervised hierarchical clustering revealed distinct metabolic patterns of monocytes recovered from CT and CTO treated mice (Fig 4C and 4D). Pathway impact analysis of differentially expressed metabolites revealed that arginine, proline, and branched chain amino acid metabolism were most significantly impacted (Fig 4E). Despite these significant pathway changes, analysis of individual metabolites only identified a few significant differences, including a trend towards decreased NAD^+^ levels in monocytes recovered from CTO treated animals (Table 2), which is indicative of OxPhos inhibition by oligomycin.

**Table 2:** Intracellular metabolites from CT and CTO treated MDSCs and monocytes*.

	MDSCs				Monocytes			
	CT	CTO	Fold Δ	*p*-value	CT	CTO	Fold Δ	*p*-value
1,4-diaminobutane	1669.81	575.51	0.3447	**0.0086**	1991.87	2058.02	1.0332	0.9245
betaine aldehyde	11707.97	2537.86	0.2168	**0.0104**	9938.89	8862.38	0.8917	0.7446
choline	2217322.04	531963.86	0.2399	**0.0055**	2020691.48	1739095.78	0.8606	0.5847
dimethyglycine	741416.78	176288.74	0.2378	**0.0065**	680422.89	576081.09	0.8467	0.5455
amino isobutyrate	15117.84	2921.84	0.1933	**0.0039**	13398.75	9508.84	0.7097	0.2922
beta hydroxybutyrate	993.14	127.41	0.1283	**0.0140**	550.54	375.74	0.6825	0.5021
serine	27945.98	6009.19	0.2150	0.2598	25013.70	17083.78	0.6830	0.5652
creatinine	1267382.01	224150.40	0.1769	**0.0088**	1283060.49	1050524.89	0.8188	0.5022
proline	32227.47	6255.23	0.1941	0.1778	36241.89	17299.73	0.4773	0.0600
betaine	46961.12	12616.09	0.2686	**0.0050**	28747.46	23328.76	0.8115	0.5316
valine	30293.38	5851.79	0.1932	**0.0029**	31134.19	26913.39	0.8644	0.6077
indole	463265.15	116725.17	0.2520	**0.0002**	470724.70	339231.95	0.7207	0.3317
nicotinamide	148691.12	22747.25	0.1530	**0.0013**	92465.86	106831.39	1.1554	0.7327
mesaconic acid	3138832.39	902198.18	0.2874	**0.0029**	2613194.80	2171897.97	0.8311	0.4744
itaconic acid	418700.34	120019.94	0.2866	**0.0028**	377928.56	302611.59	0.8007	0.3956
pipecolic acid	1367307.32	300066.48	0.2195	**0.0068**	1346786.74	1164982.29	0.8650	0.5991
methyl-oxo-pentanoate	4295.21	1879.28	0.4375	0.1140	3283.94	1856.03	0.5652	**0.0493**
leucine	762747.01	168025.76	0.2203	**0.0101**	681946.44	619007.47	0.9077	0.7277
creatine	2184525.87	477143.44	0.2184	**0.0075**	1924037.06	1500330.74	0.7798	0.3511
ornithine	181320.30	35614.43	0.1964	**0.0047**	137901.12	127836.07	0.9270	0.7911
aspartate	43571.45	14625.59	0.3357	**0.0041**	39461.81	30715.02	0.7783	0.3703
methylcysteine	162442.78	34853.59	0.2146	**0.0035**	141086.24	127112.91	0.9010	0.7304
methylnicotinamide	15618.70	4716.50	0.3020	**0.0175**	11210.60	10691.12	0.9537	0.8729
hypoxanthine	475165.00	80617.09	0.1697	**0.0068**	487605.85	391813.14	0.8035	0.4420
spermidine	1068.74	297.65	0.2785	**0.0219**	2486.46	1862.20	0.7489	0.3961
lysine	516459.23	106913.54	0.2070	**0.0059**	515826.83	448887.04	0.8702	0.6085
glutamine	60450.16	12879.45	0.2131	**0.0138**	56231.97	47633.94	0.8471	0.6012
glutamate	1222459.00	269634.98	0.2206	**0.0056**	1260582.55	1048365.28	0.8317	0.5155
acetylserine	409522.99	90174.14	0.2202	**0.0031**	420870.73	361506.78	0.8589	0.6131
amino octanoic acid	416166.30	214208.79	0.5147	**0.0197**	198258.16	166393.41	0.8393	0.5679
carnitine	143660.50	35568.69	0.2476	**0.0078**	206066.04	133416.41	0.6474	0.1979
methionine sulfoxide	14441.79	4322.62	0.2993	**0.0287**	16613.50	16020.02	0.9643	0.9054
pyridoxine	892902.77	171808.98	0.1924	**0.0081**	729019.37	778520.79	1.0679	0.8037
methyl histidine	117630.38	24551.32	0.2087	**0.0097**	106123.92	110072.95	1.0372	0.8923
arginine	654825.55	215629.05	0.3293	**0.0089**	537270.27	496236.71	0.9236	0.7618
acetyl ornithine	427325.21	141033.61	0.3300	**0.0061**	369919.76	329268.98	0.8901	0.6792
carbamoyl aspartate	18072.10	5539.75	0.3065	**0.0010**	17379.64	11672.62	0.6716	0.2083
tyrosine	151214.76	33934.40	0.2244	**0.0037**	130431.15	123118.45	0.9439	0.8476
phosphorylcholine	54710.35	42805.98	0.7824	0.0887	59220.23	23283.73	0.3932	**0.0039**
acetylglutamine	1991829.48	351228.24	0.1763	**0.0062**	1653195.62	1708981.37	1.0337	0.9040
acetyllysine	10319.45	1532.13	0.1485	**0.0092**	11230.10	7776.12	0.6924	0.2379
acetyl glutamate	76637.80	14549.64	0.1898	**0.0046**	65764.10	70330.71	1.0694	0.8062
metanephrine	17549.15	4814.09	0.2743	**0.0022**	19088.25	17776.91	0.9313	0.8259
Ng,NG-dimethyl-L-arginine	311055.15	56018.05	0.1801	**0.0049**	305985.17	265305.75	0.8671	0.6235
acetylcarnitine	4737020.76	1980968.73	0.4182	**0.0074**	2705159.57	2617356.63	0.9675	0.9108
tryptophan	179625.10	33535.58	0.1867	**0.0046**	133199.94	137709.31	1.0339	0.9210
kyneurinine	12216.08	1884.32	0.1542	**0.0054**	7923.02	10551.98	1.3318	0.4320
cystathionine	3953.59	876.94	0.2218	**0.0401**	2903.78	2815.83	0.9697	0.9045
deoxycytidine	7338.92	10391.03	1.4159	0.1553	6806.89	5245.49	0.7706	0.5122
cytidine	300405.65	53476.29	0.1780	**0.0043**	221483.90	217334.63	0.9813	0.9560
biotin	34804.36	8173.01	0.2348	0.0614	38784.23	25758.81	0.6642	0.2101
glycerophosphocholine	311456.96	92877.93	0.2982	**0.0093**	284310.52	258921.43	0.9107	0.7302
thiamine	19104.60	3979.46	0.2083	**0.0476**	18692.29	13920.87	0.7447	0.2821
phenyl acetylglutamine	47370.51	14792.02	0.3123	**0.0060**	44993.63	30845.69	0.6856	0.3015
1-methyladenosine	40307.90	6109.45	0.1516	**0.0042**	37126.74	33451.71	0.9010	0.7631
xanthosine	85345.17	13768.11	0.1613	**0.0153**	74332.18	65463.52	0.8807	0.7029
argininosuccinate	13556.30	7281.87	0.5372	**0.0240**	13053.01	9895.81	0.7581	0.3789
S-methyl-5-thioadenosine	18507.23	22232.78	1.2013	0.2249	2298.39	873.51	0.3801	0.1382
7-methylguanosine	28175.90	5378.28	0.1909	**0.0210**	27813.06	24382.54	0.8767	0.6404
glutathione	21478.94	29878.45	1.3911	0.1023	ND	ND		
CMP	18507.23	22232.78	1.2013	0.2249	2306.05	873.51	0.3788	0.1351
nicotinamide ribotide	3356.04	3676.34	1.0954	0.5558	ND	ND		
AMP	28448.06	30887.49	1.0858	0.4732	8049.87	3738.80	0.4645	**0.0131**
IMP	11732.11	13683.55	1.1663	0.3770	1642.56	643.99	0.3921	0.2032
GMP	3931.15	4056.12	1.0318	0.8329	1118.19	701.40	0.6273	0.3903
S-adenosyl-L-homocysteine	4002.15	2406.47	0.6013	0.0563	3019.71	2565.31	0.8495	0.6641
glutathione disulfide	20744.17	20852.46	1.0052	0.9661	ND	ND		
NAD	50846.01	55452.07	1.0906	0.4967	2199.33	734.98	0.3342	0.0634
NADH	363.63	987.24	2.7149	**0.0042**	ND	ND		
cobalamin	42518.68	19962.08	0.4695	0.1965	26238.10	21732.47	0.8283	0.6150
NADP	13168.63	16871.25	1.2812	0.2944	1681.04	702.57	0.4179	0.2328
flavin adenine dinucleotide	1776.41	1178.30	0.6633	0.2236	ND	ND		
lactate	14980.56	3222.05	0.2151	**0.0230**	16907.24	12507.33	0.7398	0.2566
succinate	7680.03	1343.77	0.1750	**0.0419**	5581.73	4892.55	0.8765	0.6752
methylmalonic acid	8013.86	1210.10	0.1510	**0.0104**	5162.45	6284.79	1.2174	0.5080
taurine	19805.61	20370.96	1.0285	0.8455	2644.42	2371.29	0.8967	0.7719
malate	7641.33	2564.13	0.3356	**0.0088**	6923.16	7305.33	1.0552	0.8244
hypoxanthine	3681.43	448.85	0.1219	**0.0185**	4291.35	3793.19	0.8839	0.6975
anthranilate	3867.51	824.43	0.2132	**0.0124**	3144.91	2289.36	0.7280	0.4663
phenylpropiolic acid	1214.54	198.35	0.1633	**0.0184**	816.78	786.69	0.9632	0.9399
2-hydroxygluterate	1178.11	258.93	0.2198	**0.0292**	551.86	684.59	1.2405	0.5719
xanthine	32943.83	17517.83	0.5317	0.1727	77590.44	57480.76	0.7408	0.5022
allantoin	8097.74	1299.27	0.1604	**0.0006**	7292.10	8242.82	1.1304	0.7087
phenyllactic acid	257.84	170.87	0.6627	0.4469	197.70	157.34	0.7958	0.7257
phosphoenolpyruvate	224.91	363.94	1.6182	0.2645	ND	ND		
uric acid	2041.39	1362.70	0.6675	0.2037	1529.00	1838.30	1.2023	0.6861
aconitate	3470.42	701.06	0.2020	**0.0011**	4137.89	4142.85	1.0012	0.9971
pyrophosphate	344093.90	97693.23	0.2839	**0.0021**	234661.09	211191.49	0.9000	0.6907
glucose	42435.23	7658.38	0.1805	**0.0028**	33657.50	32159.00	0.9555	0.8825
myo-inositol	82324.23	14296.03	0.1737	**0.0054**	63890.10	59667.70	0.9339	0.7999
phosphoglycerate	3675.21	2552.41	0.6945	**0.0087**	2094.56	693.79	0.3312	**0.0230**
citrate	1792491.13	472481.58	0.2636	**0.0036**	1282441.02	1265529.03	0.9868	0.9590
2-dehydro-D-gluconate	3741.68	504.17	0.1347	**0.0012**	3126.74	2136.40	0.6833	0.3685
D-gluconate	5531.46	977.45	0.1767	**0.0037**	5885.43	4126.70	0.7012	0.3181
xanthurenic acid	3490.77	490.47	0.1405	**0.0125**	4869.40	5399.37	1.1088	0.7607
pantothenate	103332.69	15649.02	0.1514	**0.0093**	94030.70	86732.95	0.9224	0.7991
uridine	2709.69	257.85	0.0952	**0.0187**	2563.72	3400.09	1.3262	0.5275
hexose phosphate	4513.27	1978.78	0.4384	**0.0060**	1836.79	1041.04	0.5668	0.2546
glucose phosphate	2746.32	1759.76	0.6408	0.0659	1389.03	983.38	0.7080	0.5452
inosine	4503.24	1373.42	0.3050	**0.0010**	2542.28	2661.56	1.0469	0.9143
sedoheptulose 7 phosphate	595.62	516.02	0.8664	0.6835	ND	ND		
D-sedoheptulose-1-7-phosphate	1104.45	912.67	0.8264	0.4560	ND	ND		
geranyl pyrophosphate	4059.84	1297.46	0.3196	**0.0076**	3787.62	2600.52	0.6866	0.2682
octulose monophosphate	1597.37	652.06	0.4082	**0.0352**	1056.36	1375.83	1.3024	0.1732
ADP	1988.29	1691.72	0.8508	0.5900	ND	ND		
dGDP	1561.96	1604.88	1.0275	0.9293	ND	ND		
cholesteryl sulfate	17872.15	3857.99	0.2159	**0.0117**	18794.03	12592.39	0.6700	0.2539
ATP	1229.38	1739.12	1.4146	0.2222	ND	ND		
dGTP	3899.68	4498.80	1.1536	0.5679	ND	ND		
UDP-D-glucose	1491.79	1856.02	1.2442	0.4926	ND	ND		
UDP-N-acetyl-glucosamine	274.12	404.66	1.4762	0.2217	ND	ND		

*Fold-change values are expressed as CTO relative to CT; ND = not detected

The lack of a clearly defined glycolytic metabolite profile in monocytes following oligomycin nanoparticle treatment was not unexpected for two reasons. First, our analysis included all biofilm-associated monocytes because of limiting cell numbers, of which only a fraction had internalized nanoparticles (Fig 3D). Second, the signals that dictate cell polarization *in vivo* are complex and it is now well appreciated that MΦs are highly plastic *in vivo* and often express both pro- and anti-inflammatory attributes [25,26]. In contrast, the majority of information describing MФ metabolic states is based on *in vitro* studies where conditions are tightly controlled [34]. Therefore, it is not unreasonable to expect differences between monocyte/MФ metabolic profiles *in vivo* vs. *in vitro*, particularly when considering the myriad of signals that are produced by *S*. *aureus* biofilms and other biofilm-associated leukocytes that monocytes encounter *in vivo*.

To examine whether the shift in the monocyte metabolome and OxPhos activity in response to oligomycin translated into enhanced pro-inflammatory properties, RT-qPCR was performed on FACS-purified monocytes using a panel of pro- and anti-inflammatory genes. Monocytes isolated from CTO treated mice displayed increased pro-inflammatory (HIF-1α, iNOS, TNF-α, Trem1, IL-1β, and IL-1α) concomitant with reduced anti-inflammatory (arginase-1, IL-10, and Trem2) markers compared to monocytes exposed to control (CT) nanoparticles (Fig 4F and S6 Fig). In addition, reactive oxygen species (ROS) production was increased in monocytes following CTO treatment *in vivo* (Fig 4G), in agreement with the finding that CTO nanoparticles potentiated bactericidal activity in mouse MΦs, whereas empty (CT) nanoparticles had no effect (S1 Fig). The magnitude of metabolic and inflammatory mediator changes elicited by oligomycin nanoparticles *in vivo* were likely modest because <5% of recovered monocytes were Cy5^+^ at day 3 post-injection (Fig 3D), which further emphasizes the significance of these findings. Earlier intervals following nanoparticle injection were not examined to allow sufficient time for oligomycin to re-program monocyte metabolism following nanoparticle uptake. Collectively, these results establish that targeting oligomycin to monocytes using tuftsin nanoparticles induces a metabolic shift to attenuate OxPhos activity, which promotes their pro-inflammatory properties.

#### Monocyte targeted oligomycin nanoparticles induce metabolomic changes in MDSCs, indicative of cellular crosstalk

**Fig 5:**
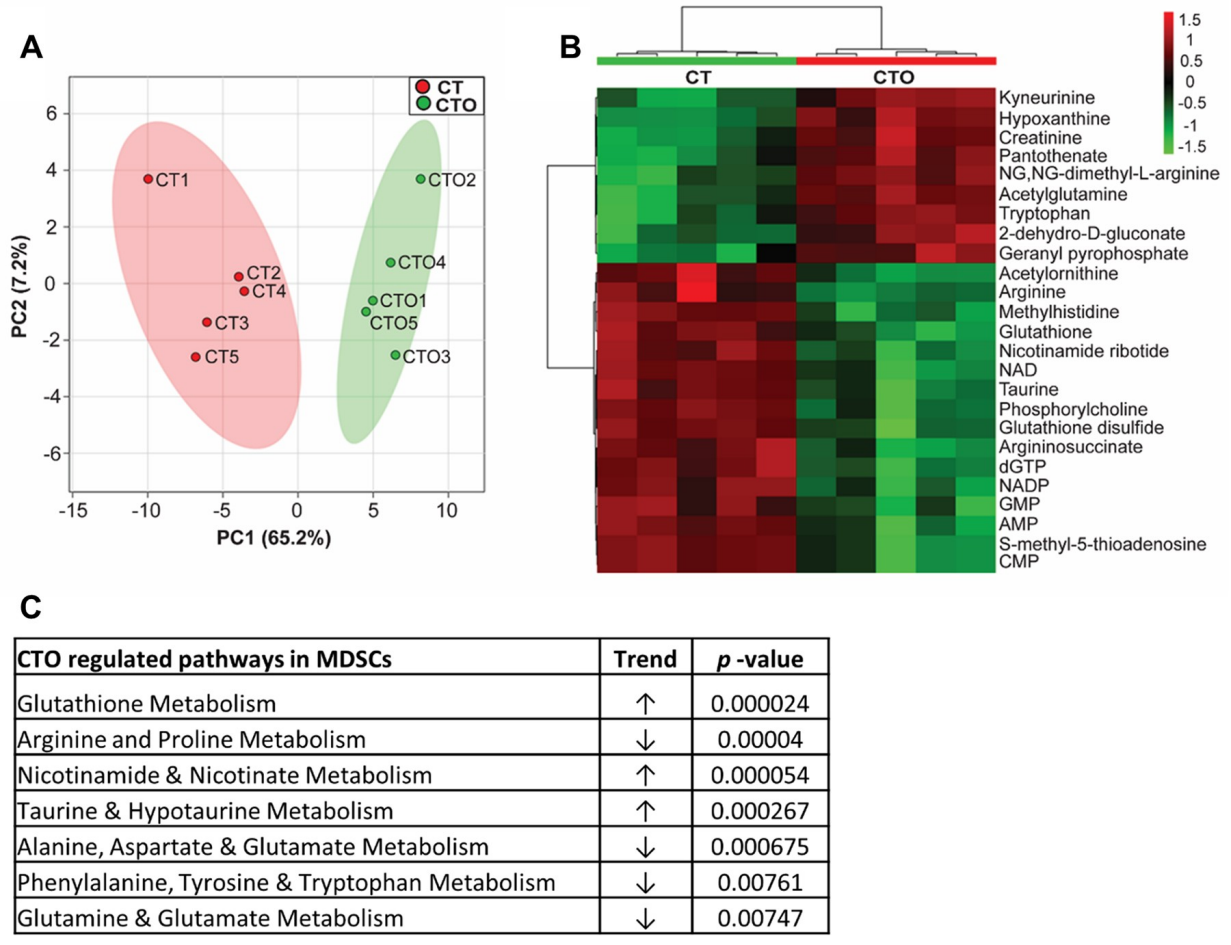
Oligomycin-containing nanoparticles targeted to monocytes induce metabolomic changes in MDSCs. C57BL/6NCrl mice received a single intra-articular injection of Cy5/Tuftsin (CT) or Cy5/Tuftsin/Oligomycin (CTO) nanoparticles (10 μg) at day 7 post-infection (n = 5 mice/treatment group). Mice were sacrificed 3 days following nanoparticle injection and MDSCs (CD11b^high^Ly6G^+^Ly6C^+^F4/80^-^) were recovered from each individual animal by FACS, whereupon intracellular metabolites were isolated for analysis by HPLC-MS/MS. (**A**) A principle component analysis (PCA) plot was generated using an algorithm in MetaboAnalyst with mean intensities and pareto scaling distribution. Ellipses represent a 95% confidence interval of the normal distribution for each cluster. (**B**) The heat map depicts the top 25 metabolite differences in MDSCs recovered from CTO and CT treated mice. The color key indicates log_2_-fold changes of normalized mean peak intensities for metabolites in MDSCs from CTO vs. CT nanoparticle treated animals. (**C**) Identification of the most significantly altered pathways in MDSCs isolated from CTO treated mice as determined by pathway impact analysis.

Prior studies have identified a significant role for MDSCs in promoting monocyte anti-inflammatory activity during *S*. *aureus* orthopedic biofilm infection [12–14]. Although <0.04% of MDSCs internalized tuftsin nanoparticles *in vivo* (Fig 3D), we were interested in determining the degree of crosstalk between metabolically altered monocytes and MDSCs. MDSCs (CD11b^high^Ly6C^+^Ly6G^+^F4/80^-^) were purified from CT and CTO treated animals, whereupon metabolomics was performed. Remarkably, although nanoparticle uptake was negligible in MDSCs, their metabolic profile was dramatically affected by CTO treatment (Fig 5A and 5B), with a number of significant differentially expressed metabolites (Table 2). Metabolic pathway analysis revealed significant increases in glutathione, nicotinamide, and taurine metabolism, whereas arginine, glutamine, and cyclic chain amino acids were significantly reduced (Fig 5C). To the best of our knowledge, these results are the first to demonstrate that metabolic reprogramming of monocytes can impact MDSC metabolism, which supports our earlier work of MDSC-monocyte crosstalk during biofilm infection [12–14].

#### Nanoparticle-mediated delivery of oligomycin to monocytes attenuates established *S. aureus* PJI

**Fig 6:**
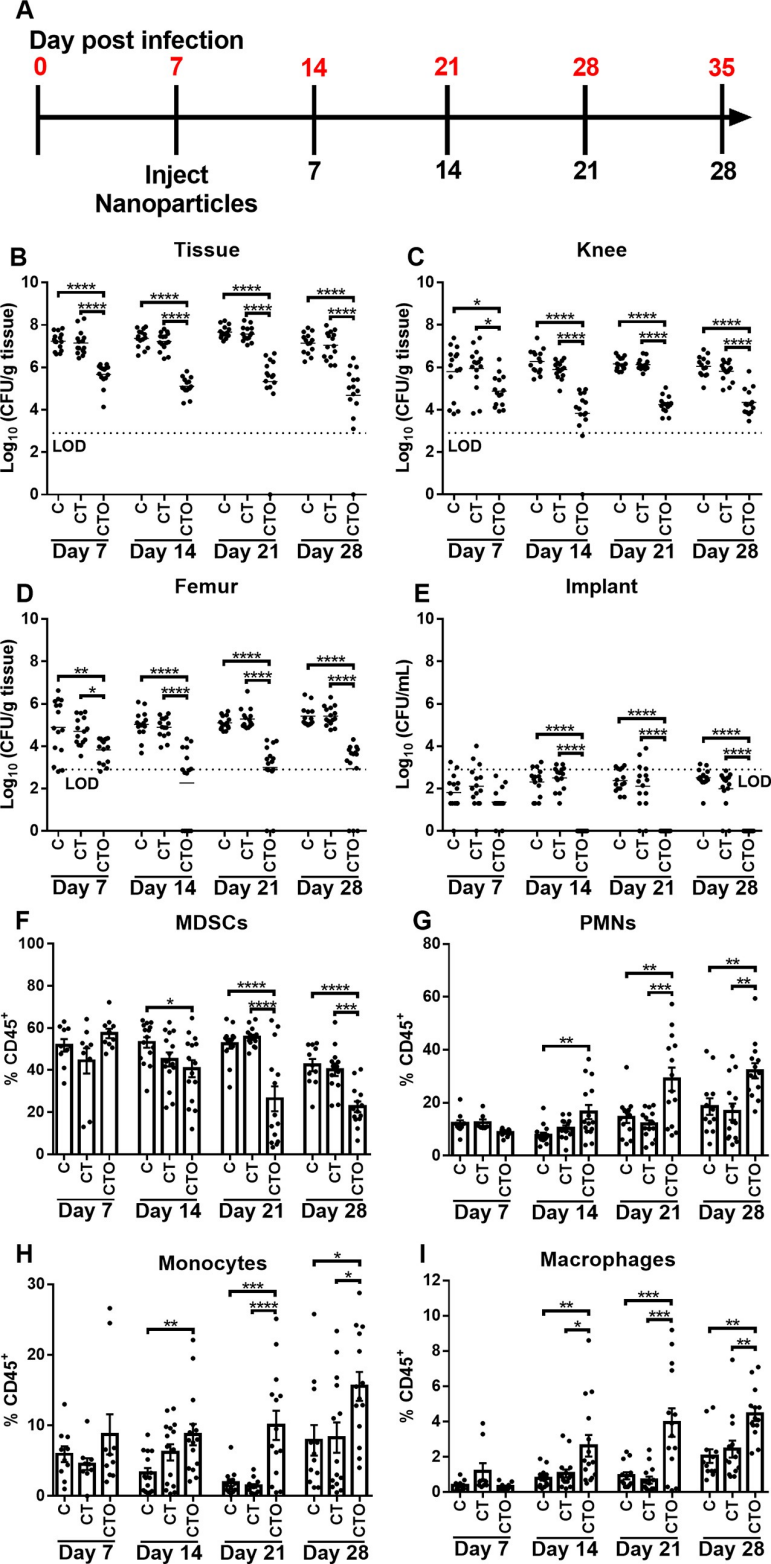
Nanoparticle-mediated delivery of oligomycin to monocytes reduces established biofilm infection. (**A**) C57BL/6NCrl mice received a single intra-articular injection of Cy5 (C), Cy5/Tuftsin (CT), or Cy5/Tuftsin/Oligomycin (CTO) nanoparticles at day 7 post-infection and were analyzed out to 28 days following nanoparticle treatment. Bacterial burden was quantified in the (**B**) surrounding soft tissue, (**C**) knee, (**D**) femur, and (**E**) implant, where the dotted line represents the limit of detection (LOD). Infiltrating leukocytes were analyzed by flow cytometry and (**F**) MDSCs, (**G**) PMNs, (**H**) monocytes, and (**I**) macrophages are reported as the percentage of live CD45^+^ leukocytes (mean ± SD). Results are combined from three independent experiments (n = 15 mice/group/time point). (*, *p* < 0.05; **, *p* < 0.01; ***, *p* < 0.001; ****, *p* < 0.0001; One-way ANOVA).

We next examined whether the ability of oligomycin nanoparticles to reprogram monocyte pro-inflammatory properties would translate into improved biofilm clearance during *S*. *aureus* PJI. Two treatment paradigms were designed to examine the effect of monocyte metabolic reprogramming during acute tissue infection versus an established biofilm (days 3 and 7 post-infection, respectively). Mice received a single intra-articular injection of control (C and CT) or oligomycin (CTO) nanoparticles on either day 3 (S7 Fig) or day 7 post-infection (Fig 6), whereupon bacterial burden and leukocyte infiltrates were examined over a 28 day period. In both settings, only CTO nanoparticles significantly reduced bacterial burden in the soft tissue, joint, femur, and implant between days 7 and 28 following injection (S7 Fig and Fig 6B–6E). Interestingly, despite the reductions in bacterial titer at day 7 with CTO nanoparticles in both treatment paradigms, no significant differences in MDSC (CD11b^high^Ly6C^+^Ly6G^+^F4/80^-^), PMN (CD11b^low^Ly6C^+^Ly6G^+^F4/80^-^), monocyte (CD11b^high^Ly6C^+^Ly6G^-^F4/80^-^), or MФ (CD11b^high^Ly6C^-^Ly6G^-^F4/80^+^) infiltrates were detected at this time point (S7 Fig and Fig 6F–6I). Therefore, the observed reduction in bacterial burden at day 7 may result from enhanced bactericidal activity of biofilm-associated monocytes following oligomycin treatment, since cells exhibited increased pro-inflammatory mediator expression that typically coincides with enhanced ROS production [39,40]. Indeed, we found that oligomycin enhanced MФ killing of *S*. *aureus* and ROS production (Figs S1 and 4G, respectively). At later time points (i.e. days 14 to 28 after nanoparticle treatment) mice receiving CTO nanoparticles had significantly fewer MDSCs concomitant with increased PMN, monocyte, and MФ infiltrates (Fig 6 and S7F–S7I Fig), a relationship that our prior studies have established is reflective of a more pro-inflammatory milieu and is associated with biofilm clearance [12–14].

To confirm that the ability of oligomycin nanoparticles to attenuate *S*. *aureus* biofilm burden resulted from monocyte metabolic reprogramming rather than direct antibacterial activity, mice were treated with free oligomycin only, empty nanoparticles (CT) with free oligomycin (not loaded), or oligomycin loaded nanoparticles (CTO). Free oligomycin was administered at a dose that was equivalent to the nanoparticle formulation (100 ng). Only CTO-containing nanoparticles were capable of reducing biofilm burden (S8A Fig), which was first evident at day 7 post-injection, in agreement with our earlier findings (Fig 6 and S5 and S7 Fig). Importantly, no changes in biofilm burden were observed in animals treated with free oligomycin or CT nanoparticles + free oligomycin (not loaded) at either 3 or 7 days after intra-articular injection (S8A Fig). Furthermore, oligomycin delivery by direct intra-articular injection into the infected joint in either one bolus or two sequential doses also had no effect on bacterial burden (S8B Fig). Oligomycin also lacked bactericidal activity against *S*. *aureus* across a range of concentrations during planktonic growth, early biofilm formation, and mature biofilm *in vitro* (S9 Fig), in agreement with prior reports demonstrating that oligomycin alone does not affect *S*. *aureus* growth [41,42]. To rule out the possibility that oligomycin altered *S*. *aureus* metabolism to make organisms more susceptible to immune-mediated clearance, metabolomics was performed. Oligomycin had minimal effects on *S*. *aureus* metabolism, since of the 170 intracellular metabolites detected; only 14 or 19 were significantly altered with oligomycin treatment during biofilm or planktonic growth, respectively (S10 Fig). In addition, oligomycin did not affect *S*. *aureus* glycolysis, since glycolytic intermediates were not altered following oligomycin treatment (S11 Fig). Finally, we were unable to detect free oligomycin in implant-associated tissue at day 3 following CTO nanoparticle injection by HPLC, whereas a spiked derivative of oligomycin (5 ng) was evident (S12 Fig). We chose to evaluate levels at day 3 post-injection, since this was expected to reflect maximal extracellular levels of oligomycin if burst release had occurred, as established by the kinetics of drug release *in vitro* (S2A Fig). These results demonstrate that the amount of free oligomycin in the joint was less than 5 ng per 50 mg of tissue (based on the sensitivity of detecting the spiked oligomycin derivative). Collectively, these findings establish that oligomycin leaching from nanoparticles *in vivo* is negligible and argues against major effects on *S*. *aureus* metabolism, since few metabolic changes occurred in biofilms with a concentration of free oligomycin (10 μg/ml) that is over 2000-fold higher than levels associated with the joint *in vivo*. Taken together, these results establish that the anti-biofilm effects of oligomycin are dependent on targeted intracellular delivery to monocytes and their subsequent metabolic reprogramming to elicit pro-inflammatory activity, and not as an antibiotic or via changes in *S*. *aureus* metabolism.

#### Combined action of oligomycin nanoparticles and systemic antibiotics to clear established *S. aureus* biofilm infection

**Fig 7:**
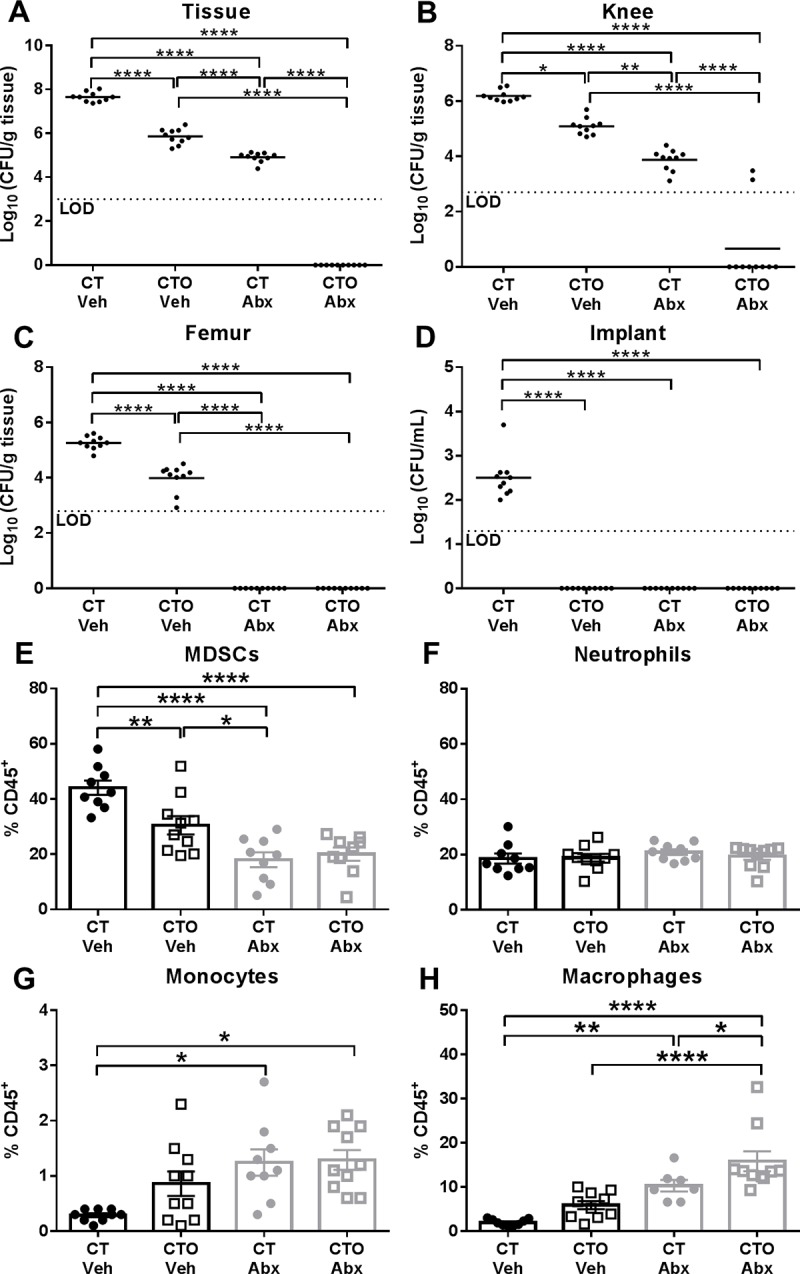
Monocyte metabolic reprogramming with oligomycin-containing nanoparticles cooperates with antibiotics to clear established *S*. *aureus* biofilm infection. C57BL/6NCrl mice received a single intra-articular injection of Cy5/Tuftsin (CT) or Cy5/Tuftsin/Oligomycin (CTO) nanoparticles (10 μg) at day 7 post-infection. Seven days later, animals received daily i.p. injections of antibiotics (Abx; 25 mg/kg/day rifampin and 5 mg/kg/day daptomycin) or vehicle (Veh) for one week, whereupon mice were sacrificed at day 21 post-infection. Bacterial burden was quantified from the (**A**) surrounding soft tissue, (**B**) knee, (**C**) femur, and (**D**) implant, where the dotted line represents the limit of detection (LOD). Infiltrating leukocytes were analyzed by flow cytometry and (**E**) MDSCs, (**F**) neutrophils, (**G**) monocytes, and (**H**) macrophages are reported as the percentage of live CD45^+^ leukocytes (mean ± SD). Results are combined from two independent experiments (n = 7–10 mice/group/time point). (*, *p* < 0.05; **, *p* < 0.01; ****, *p* < 0.0001; One-way ANOVA).

We next examined whether the residual bacteria present following oligomycin nanoparticle treatment may have increased susceptibility to systemic antibiotics based on the immune transformation of the biofilm milieu to a pro-inflammatory state. To address this possibility, mice received CT or CTO nanoparticles on day 7 post-infection, whereupon systemic antibiotics (25 mg/kg/day rifampin and 5 mg/kg/day daptomycin) were administered 7 days later for one week after which mice were sacrificed (corresponding to day 21 post-infection). Remarkably, bacterial burden was largely below the limit of detection in animals that received CTO nanoparticles and antibiotics (Fig 7A–7D). These changes were reflected by significant reductions in MDSCs and increased monocyte and MΦ recruitment (Fig 7E, 7G & 7H), which our results show are reprogrammed towards a pro-inflammatory phenotype by intracellular oligomycin delivery (Fig 4F and 4G and S6 Fig). In contrast, bacteria remained in the tissue and knee of mice treated with control CT nanoparticles and antibiotics or CTO nanoparticles alone (Fig 7A and 7B). The ability of systemic antibiotics to lower bacterial counts in the femur and implant of animals receiving control CT nanoparticles (CT Abx) to below the limit of detection likely resulted from the high doses of daptomycin and rifampin that were used, which have been shown by others to exhibit anti-biofilm activity *in vivo* [43–46]. In addition, bacterial burden in the femur and implant was not as robust compared to the knee joint and surrounding tissue, which may make the former more susceptible to antibiotic-mediated clearance. Indeed, a prior study demonstrated that daptomycin (10 mg/kg) reduced *S*. *aureus* burden on orthopedic implants to near the limit of detection (i.e. ~ < 10^2^ CFU) in the same mouse PJI model, whereas titers in the joint were less affected [47]. Importantly, daptomycin and rifampin were not capable of clearing infection in the tissue or joint unless combined with oligomycin nanoparticles (Fig 7A and 7B), demonstrating the need for concurrent monocytic metabolic reprogramming. In a separate study, mice were subjected to the same treatment paradigm but were sacrificed at day 28, which confirmed the superior combination of oligomycin-containing nanoparticles (CTO) and systemic antibiotics (S13 Fig). The ability of systemic antibiotics to clear infection with oligomycin nanoparticle treatment is attributed to the immune-mediated effects of oligomycin on monocyte metabolism and not by altering *S*. *aureus* metabolism to enhance antibiotic susceptibility, since oligomycin had minimal effects on the *S*. *aureus* metabolome under both biofilm and planktonic growth conditions (S10 Fig and S11 Fig). To our knowledge, this is the first demonstration of an approach capable of clearing an established *S*. *aureus* biofilm infection, which was achieved by modulating monocyte metabolism to enhance *S*. *aureus* biofilm antibiotic susceptibility.

## Discussion

**Fig 8:**
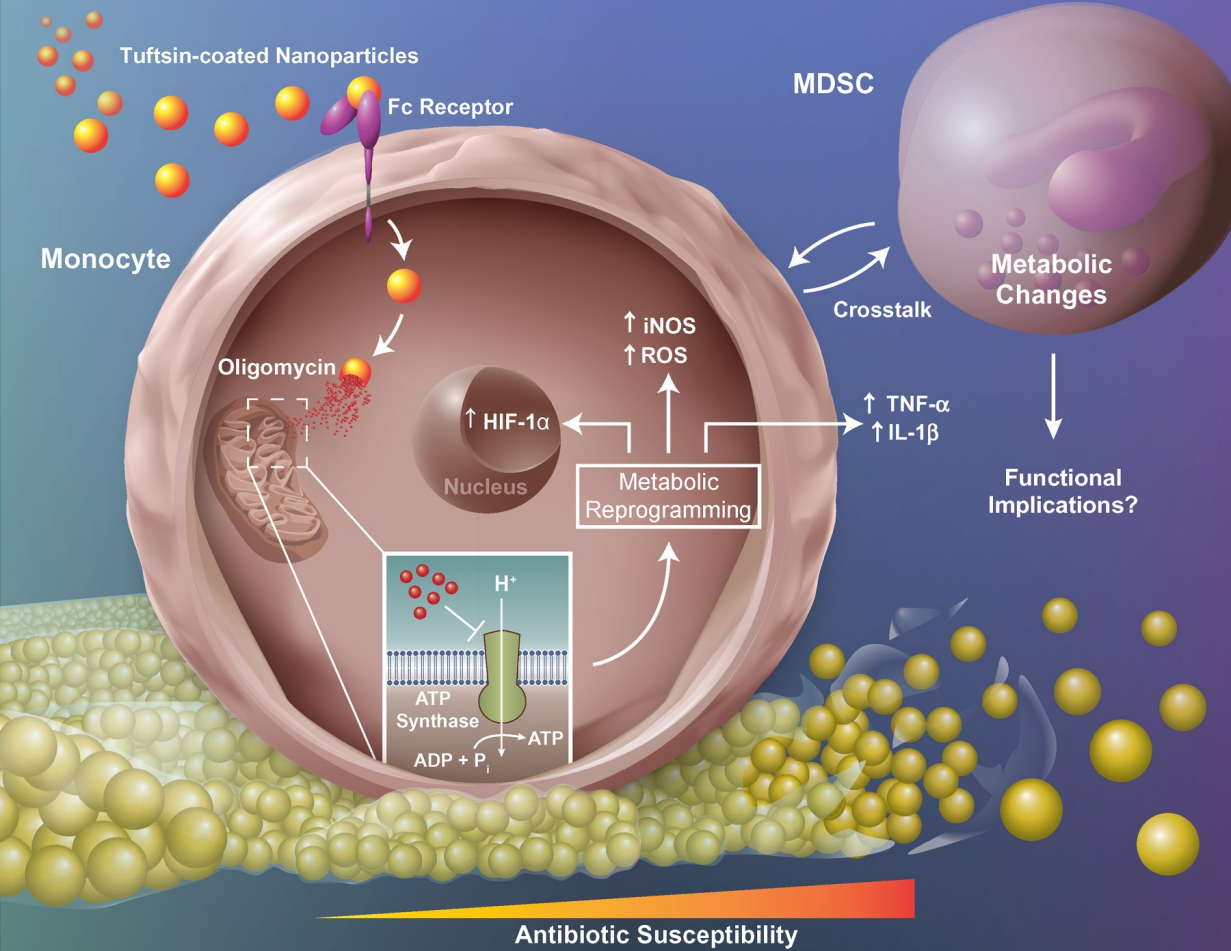
Metabolic reprogramming of monocytes promotes pro-inflammatory properties, MDSC crosstalk, and *S*. *aureus* biofilm clearance. Nanoparticles containing the ATP synthase inhibitor oligomycin are targeted to Fc-receptor positive monocytes with tuftsin. Upon internalization, oligomycin inhibits ATP synthase of the mitochondrial electron transport chain and induces metabolic reprogramming to shift monocyte metabolism and promote pro-inflammatory gene expression. Metabolically reprogrammed monocytes also influence MDSC metabolism. Collectively, these changes promote increased antibiotic susceptibility and clearance of established biofilm infection. ADP, adenosine diphosphate; ATP, adenosine triphosphate; Fc-Receptor, fragment crystallizable region receptor; HIF-1α, hypoxia-inducible factor 1-alpha; IL-1β, interleukin-1β; iNOS, inducible nitric oxide synthase; MDSC, myeloid-derived suppressor cell; P_i_, inorganic phosphate; ROS, reactive oxygen species; TNF-α, tumor necrosis factor-α.

Approximately one million knee and hip arthroplasties are performed in the United States annually, with infection representing the most common complication [1,5,48,49]. PJIs are associated with significant morbidity and medical expenses, since the current standard-of-care requires multiple surgical interventions over a period of months, during which patient mobility is limited [3–5]. *S*. *aureus* is a common cause of PJI [5,48,49] and biofilms cannot typically be cleared by antibiotics without removing the prosthesis, since the metabolic dormancy of a subpopulation of biofilm-associated bacteria decreases their antibiotic susceptibility [5,7,50]. Further compounding this issue is the ability of *S*. *aureus* to promote an anti-inflammatory milieu typified by the polarization of anti-inflammatory monocytes and abundance of MDSC infiltrates [12–15]. Monocytes can be key effector cells during PJI in a permissive microenvironment, such as during MDSC depletion, where their pro-inflammatory activity is augmented leading to reduced biofilm burden [14]. This study is the first to demonstrate that biofilm-associated monocytes are metabolically skewed towards OxPhos to support their anti-inflammatory properties. Interestingly, monocyte OxPhos activity was significantly lower during the first two days of *S*. *aureus* infection compared to sterile implants, whereas cells transitioned to favor OxPhos at later stages (i.e. days 5–7). These findings coincide with the kinetics of biofilm development *in vivo*, where our prior studies have shown that biofilm maturation is evident by day 7 post-infection as revealed by antibiotic tolerance [15]. By extension, the early reduction in OxPhos activity suggests that monocytes may initially exhibit pro-inflammatory properties but transition to an anti-inflammatory phenotype that we have associated with mature biofilms [12–15]. This potential early pro-inflammatory activity may explain the conundrum of heightened pro-inflammatory mediator production at the site of biofilm infection, which has limited impact on the intrinsic anti-inflammatory properties of biofilm-associated leukocytes or biofilm burden [12–15]. Furthermore, this study is the first to demonstrate that biofilm-associated monocytes can be metabolically reprogrammed to inhibit OxPhos, transforming cells into a pro-inflammatory state capable of attenuating established *S*. *aureus* biofilms (Fig 8).

We selected oligomycin to reprogram monocytes in this study over other metabolic inhibitors because it is less potent and would dampen but not completely block OxPhos, whereas stronger inhibitors, such as rotenone, are extremely toxic and have been shown to inhibit MΦ viability and function [51–54]. In addition, a nanoparticle-directed method was preferred for *in vivo* administration because of potential toxicity associated with a non-targeted systemic delivery approach [55,56]. In this study, nanoparticles were conjugated with tuftsin to facilitate FcR-mediated uptake in monocytes [36], which are the most abundant mononuclear phagocyte population during *S*. *aureus* PJI [12–15]. We established that the tuftsin modification enhanced nanoparticle uptake by monocytes approximately 4-fold compared to non-conjugated nanoparticles and nanoparticle uptake was ~37.5-fold higher in monocytes versus biofilm-associated MDSCs and PMNs.

Importantly, oligomycin nanoparticles were capable of metabolically reprogramming biofilm-associated monocytes, which coincided with their polarization to a pro-inflammatory state, typified by heightened pro- and reduced anti-inflammatory gene expression concomitant with increased ROS production. These effects were remarkable given the finding that <5% of monocytes displayed evidence of nanoparticle uptake (i.e. Cy5^+^) by flow cytometry at the time of analysis. However, it is also possible that a greater number of monocytes had internalized nanoparticles but were no longer Cy5^+^, since fluorescence is likely quenched in the phagosome as has been reported for other fluorochromes [38]. It is unclear whether this decrease in intracellular Cy5 fluorescence can be interpreted to reflect reduced oligomycin action; however, our *in vivo* data do not support this possibility, since significant differences in monocyte metabolism, cytokine expression, and ROS production were observed at intervals following oligomycin nanoparticle treatment when intracellular Cy5 signals in monocytes were low/undetectable by flow cytometry (i.e. days 3 and 7 post-nanoparticle injection, respectively). In addition, bacterial burden remained significantly reduced out to day 28 after CTO treatment, the latest time point examined. The retention of nanoparticles in the joint tissue until day 12 post-injection, as revealed by IVIS, also suggests a potential depot for continued oligomycin action. Metabolic pathway analysis of significantly downregulated metabolites showed that arginine, proline, and branched chain amino acid metabolism were most affected in monocytes isolated from CTO treated mice. Decreased amino acid abundance may reflect increased protein synthesis to satisfy the need for metabolic intermediates during immune activation, which is a hallmark of cells undergoing aerobic glycolysis [57,58]. Citrate and succinate accumulation was expected due to a glycolytic shift induced by oligomycin nanoparticle treatment and block in TCA cycle enzymes [57–59]; however, both were less abundant in CTO monocytes. Glucose and glucose-6-phosphate were also decreased in CTO monocytes, which prior *in vitro* studies showed were increased in pro-inflammatory MΦs [60]. Nevertheless, the secondary TCA cycle metabolite 2-hydroxyglutarate [60–62] was increased in CTO monocytes, indicating an inhibition of the TCA cycle that supports a glycolytic bias. The endogenous MΦ activator uric acid and its metabolite, allantoin [63,64], were also more abundant in CTO monocytes as were methyl-malonic acid and uridine, consistent with prior *in vitro* studies of LPS stimulated monocytes with potent pro-inflammatory properties [65]. Finally, itaconate, which has recently been recognized as a key metabolite in supporting MΦ anti-inflammatory activity [66], was also reduced in monocytes following oligomycin nanoparticle treatment, in agreement with a pro-inflammatory bias.

Although these metabolite patterns do not follow a strict definition of glycolytic or OxPhos metabolism that has been described *in vitro* [57,58], JC-1 staining revealed that oligomycin nanoparticles induced a metabolic shift in biofilm-associated monocytes *in vivo* as reflected by significantly less OxPhos activity, suggesting a net bias towards glycolysis. It is important to recognize that the metabolomic signatures currently defined for pro- vs. anti-inflammatory MΦs are primarily based on *in vitro* studies, where cells are polarized to activation extremes [57,58]. *In vivo*, monocytes/MΦs receive a complex array of signals from the biofilm and other leukocyte populations and exist in a spectrum of activation states, with both phenotypic and metabolic plasticity [25,26,67,68]. The mixed metabolic profile that we observed with biofilm-associated monocytes after oligomycin nanoparticle treatment *in vivo* is reminiscent of the finding that *in vivo* monocytes/MΦs often possess both pro- and anti-inflammatory attributes, in contrast to the distinct M1/M2 polarization profiles that were established by *in vitro* studies where cells were skewed to extreme activation states by well-defined stimuli (i.e. LPS vs. IL-4)[25,26]. Therefore, it is not unexpected that the metabolic profile of biofilm-associated monocytes *in vivo* possessed attributes of both OxPhos and glycolysis, which may be further explained by the milder OxPhos inhibitory activity of oligomycin as discussed earlier [27,69]. Importantly, the transformation of biofilm-associated monocytes to a pro-inflammatory state following oligomycin nanoparticle treatment was confirmed by RT-qPCR, where monocytes recovered from CTO treated animals displayed increased pro- and reduced anti-inflammatory gene expression compared to monocytes from mice receiving control nanoparticles concomitant with increased ROS production. These changes in monocyte metabolism and pro-inflammatory activity were independent of bacterial burden, which was equivalent between animals receiving oligomycin and control nanoparticles at day 3 post-treatment, and increased monocyte pro-inflammatory activity is likely responsible for the reduction in *S*. *aureus* titers from day 7 onward. Furthermore, monocyte OxPhos was unaffected following treatment with free oligomycin, establishing the requirement for intracellular delivery, which was facilitated by tuftsin-modified nanoparticles.

An unexpected finding in this study was that biofilm-associated MDSCs displayed marked alterations in their metabolome, despite that fact that <0.04% cells exhibited evidence of nanoparticle uptake. To our knowledge, this is the first demonstration of monocyte-MDSC metabolic crosstalk during biofilm infection (Fig 8), which supports our earlier observations where reduced MDSC infiltrates led to enhanced monocyte pro-inflammatory activity [12–14]. Metabolic pathway analysis revealed that MDSCs recovered from CTO treated mice had increased glutathione, nicotinamide, and taurine metabolism, whereas arginine, glutamine, negatively charged, and cyclic chain amino acids were down regulated. It is difficult to predict the impact of these metabolic changes, since few studies have explored MDSC metabolism, especially *in vivo*; however, this metabolic signature would generally be consistent with a glycolytic preference, which is in agreement with our findings that biofilm-associated MDSCs are of the granulocytic lineage (G-MDSCs) and activated PMNs are typified by robust glycolysis [15,70]. Similar to monocytes, a decrease in amino acids likely corresponds to an increase in protein synthesis or need for metabolic intermediates, which are typical attributes of glycolytic cells [57,58]. An increase in GSH/GSSH has been shown to prime MΦs and T cells for glycolytic metabolism [71,72] and the observed increase in NADH/NAD^+^ in MDSCs recovered from CTO compared to CT treated mice (2.489-fold) corresponds to a reduction in electron transport chain activity [73,74]. While elevated NADH/NAD^+^ should increase lactic acid fermentation and lactate production, intracellular lactate levels were 4-fold lower in MDSCs from CTO treated animals. One potential explanation to account for this finding is that lactate is exported from the cell by monocarboxylate transporters (MCTs), which are enhanced following immune activation [75]. The reason why MDSC metabolites were more affected compared to monocytes following CTO treatment is unclear, but may be a reflection of the lower cell yield of biofilm-associated monocytes compared to MDSCs. Nevertheless, several monocyte metabolites were altered following CTO treatment that fit the expected profile of a glycolytic bias.

Major challenges pertaining to PJI treatment are the inability to clear infection without removing the prosthesis and preventing infection recurrence. Based on our findings that oligomycin nanoparticles augmented monocyte pro-inflammatory activity, we examined how this influenced biofilm burden. Remarkably, *S*. *aureus* titers were significantly reduced in mice receiving oligomycin nanoparticles out to 28 days post-injection. This was accompanied with a reduction in MDSCs concomitant with increased monocyte, PMN, and MΦ infiltrates, which was likely responsible for biofilm clearance based on our prior studies showing that the balance between MDSCs vs. monocytes/MΦs and PMNs dictates biofilm persistence [12–14]. In addition, CTO nanoparticles enhanced ROS production in biofilm-associated monocytes, a potent antimicrobial effector. These findings are significant for several reasons. First, only a single injection of CTO nanoparticles was required reflecting sustained action, which is a desirable feature for a potential therapeutic. Second, nanoparticle treatment was not initiated until a mature biofilm was established (i.e. day 7 post-infection), which represents the most challenging treatment scenario. Third, combined treatment with CTO nanoparticles and systemic antibiotics reduced infectious burden to below the limit of detection. Importantly, we provide several lines of evidence demonstrating that oligomycin lacks direct activity against *S*. *aureus*, including the failure of free oligomycin to alter biofilm burden *in vitro* or *in vivo* and minimal effects on *S*. *aureus* metabolism under both planktonic and biofilm growth conditions. To our knowledge, this is the first demonstration of an *in vivo* approach that is capable of clearing established *S*. *aureus* biofilms without removal of the infected implant.

Understanding the complex and dynamic interactions between *S*. *aureus* biofilm and the host immune response is critical to developing new therapeutic approaches to reduce the morbidity associated with PJI. This study establishes that biofilm-associated monocytes can be metabolically reprogrammed using a nanoparticle targeted approach to promote their pro-inflammatory properties, which results in biofilm clearance. A similar strategy might represent a therapeutic for PJI by modulating host immunity to facilitate bacterial susceptibility to systemic antibiotics and alleviate the need for removal of the infected prosthesis, resulting in less patient morbidity.

## Materials and methods

### Mice

Male and female C57BL/6NCrl mice (RRID:IMSR_CRL:27; 8 weeks of age) were purchased from Charles River Laboratories (Frederick, MD). When animals were designated for experiments, mice of the same sex were randomized into standard density cages (5 animals per cage). Mice were housed in a restricted-access BSL2 room equipped with ventilated microisolator cages and maintained at 21°C under a 12 h light:12 h dark cycle with ad libitum access to water (Hydropac; Lab Products, Seaford, DE) and Teklad rodent chow (Harlan, Indianapolis, IN) with Nestlets provided for enrichment. This study was conducted in strict accordance with the ARRIVE guidelines and recommendations in the *Guide for the Care and Use of Laboratory Animals* of the National Institutes of Health. The animal protocol was approved by the Institutional Animal Care and Use Committee of the University of Nebraska Medical Center (#18-013-03).

### Mouse model of *S. aureus* orthopedic implant biofilm infection

To model infectious complications in patients following arthroplasty, a mouse model of *S*. *aureus* orthopedic implant infection was used as previously described [12,14]. This model reflects biofilm growth as demonstrated by us and others using SEM and H&E staining [13,14,47,76–78]. In addition, immunophenotyping of patients with PJI, many of which were diagnosed with *S*. *aureus*, has revealed similar leukocyte infiltrates as observed in the mouse model [13,22], supporting its translational utility. Briefly, mice were anesthetized with ketamine/xylazine (100 mg/kg and 5 mg/kg, respectively) and the skin was disinfected with povidone-iodine. A medial incision was created through the quadriceps with lateral displacement to access the distal femur. A burr hole was created in the femoral intercondylar notch using a 26-gauge needle to facilitate the insertion of a pre-cut 0.8 cm orthopedic-grade Kirschner wire (0.6 mm diameter, Nitinol [nickel-titanium]; Custom Wire Technologies, Port Washington, WI) into the intramedullary canal, leaving ~1 mm protruding into the joint space. The exposed wire was inoculated with 10^3^ CFU of *S*. *aureus* USA300 LAC13c [11] in 2 μL of PBS. The skin was closed with absorbable nylon suture and mice received s.c. Buprenex (0.1 mg/kg; Reckitt Benckiser, Hull, U.K.) for the first 24 h after surgery for pain relief. Animals were closely monitored throughout the course of infection and displayed normal behaviors and ambulation. There were no remarkable changes in animal appearance during the course of infection, other than an initial weight loss during the first two days post-surgery, which was attributed to anesthetic/post-surgical effects since these changes have also been observed in mice that receive sterile implants. Importantly, no adverse effects following oligomycin nanoparticle treatment were observed, which was not unexpected, since the compound was delivered directly into the infected joint. At the indicated time points post-infection, mice were euthanized using an overdose of inhaled isoflurane with cervical dislocation as a secondary method to ensure death.

### Primary leukocyte populations

MΦs were expanded from the bone marrow of both male and female C57BL/6NCrl mice as previously described [79] and PMNs were recovered from the peritoneal cavity of mice 24 h after injection of sterile thioglycollate and purified using an anti-Ly6G MicroBead Kit (Cat #130-092-332; Miltenyi Biotec) per the manufacturer’s instructions. Human monocytes were obtained from healthy donors by the UNMC Elutriation Core Facility using countercurrent centrifugal elutriation, in full compliance and approval of the Institutional Review Board (IRB). Human monocytes were cultured at 10^6^ cells/mL in RPMI-1640 supplemented with recombinant human M-CSF (100ng/mL; Cat #574802; BioLegend), 10% human serum (Cat #H4522; Sigma-Aldrich), and penicillin/streptomycin/fungizone for 7 days to drive macrophage differentiation.

Mouse and human MΦs were seeded at 5x10^4^ cells/well in a 96-well plate for experiments. Following an overnight adherence period, MΦs were pre-treated with 10 ng/mL recombinant mouse IL-4 (Cat #574306) or human IL-4 (Cat #574002) both from BioLegend, San Diego, CA) for 1 h followed by exposure to various concentrations of oligomycin (Cat #11342 Cayman Chemical) for 24 h, whereupon medium was collected to quantify TNF-α by cytometric bead array (mouse; Cat #552364; human, Cat #551811; both from BD Biosciences), IL-10 expression by ELISA (mouse DuoSet Cat #DY417-05, R&D Systems) or cytometric bead array (human; Cat #551811, BD Biosciences), arginase activity (Cat #MAK112, Millipore Sigma) and CD86 (mouse; Cat #105012, RRID:AB_493342; human, Cat #305438, RRID:AB_2564164; both from BioLegend) and CD204 (mouse; Cat #748085, BD Biosciences; human, Cat #371906, RRID:AB_2650770, BioLegend) expression by flow cytometry. Mouse PMNs were treated with various concentrations of oligomycin for 2 h, whereupon TNF-α, IL-10, and arginase activity were assessed.

### Nanoparticle synthesis and characterization

Polymeric nanoparticles based on an amphiphilic block copolymer of poly(ethylene glycol)-*b*-poly(L-glutamic acid) (PEG-*b*-PGA) with pendant phenylalanine functionalities were synthetized as previously described [80,81]. Briefly, PEG-*b*-PGA (Alamanda Polymers, Inc., Madison, AL; block lengths of 114 and 150 repeating units for PEG and PGA, respectively) was first modified with L-phenylalanine methyl ester via carbodiimide chemistry. The degree of grafting was 50% as determined by ^1^H-NMR analysis. Polymeric micelles were then prepared by mixing the copolymer solution in dimethylformamide with water (1:1 v/v) followed by dialysis against water for 48 h. The formed micelles were cross-linked using 1,2-ethylenediamine in the presence of 1-(3-dimethylaminopropyl)-3-ethylcarbodiimide hydrochloride (EDC) with a targeted cross-link density of 20% (based on the molar ratio of cross-linker to carboxylic groups of the GA residues). Tuftsin peptide with a cysteine residue at the C-terminus (TKPRC) was synthesized on an automated solid-phase Liberty microwave peptide synthesizer (CEM, Matthews, NC) employing standard Fmoc chemistry using a Rink Amide resin (Nova Biochem). Sample purification (≥ 95%) was performed on a Phenomenex (Torrance, CA) Jupiter 10 μm Proteo 250 × 4.6 mm C12 column using a water (0.1% formic acid)–acetonitrile (0.1% formic acid) gradient. HPLC/MS analyses were performed on a Waters (Milford, MA) e2695 system equipped with a Waters 2489 absorption detector and a Waters Q-Tof Micro electrospray ionization mass spectrometer.

Tuftsin targeting moieties were conjugated to nanoparticles via a heterobifunctional maleimide-PEG-amine linker (MAL-PEG-NH_2_, 7.5 kDa, JenKem Technology, Plane TX). First, MAL-PEG-NH_2_ (0.27 μmol, 0.15 eq with respect to the amount of carboxylic groups) was conjugated to the free carboxyl groups (1.75 μmol) of EDC-activated nanoparticles. Resulting constructs were purified using repeated ultrafiltration (MWCO 30,000, Millipore) at 2200 rpm for 15 min (3 washes). Purified peptide (0.31 μmol) was subsequently reacted with MAL-PEG functionalized nanoparticles in PBS at pH 7 for 2 h. Unreacted MAL groups were quenched by β-mercaptoethanol and targeted nanoparticles were purified by dialysis against distilled water using a dialysis membrane (MWCO 3.5 kDa). The amount of peptide conjugated on the surface of the nanoparticles was 126.3 ± 6.3 μg per mg of polymer (n = 3) as determined by a BCA protein assay. Oligomycin-loaded nanoparticles were prepared by adding an ethanol solution of oligomycin (2 mg/mL; Cat #11342 Cayman Chemical, Ann Arbor, MI) dropwise into the aqueous dispersion of tuftsin-coated nanoparticles (1 mg/mL) and mixed overnight at RT in an open-air system to allow for the slow evaporation of ethanol, whereupon residual ethanol was removed at reduced pressure. Unincorporated oligomycin was removed by filtration with 0.8 μm syringe filters (ThermoScientific). Oligomycin content was determined by HPLC analysis under isocratic conditions using an Agilent 1200 HPLC system with a diode array detector set at 226 nm [82]. A Nucleosil C18 column was used as stationary phase (250 mm × 4.6 mm), and the mobile phase was comprised of an acetonitrile/water mixture (80/20, v/v) applied at a flow rate of 1 mL/min. The resulting nanoparticles had an average hydrodynamic diameter of approximately 80 nm (ζ-potential = -8.4 mV) and were characterized by narrow size distributions using dynamic light scattering (Zetasizer Nano ZS, Malvern Instruments Ltd., Worcestershire, UK). The loading capacity of tuftsin-coated nanoparticles for oligomycin was 5.2 ± 0.9% (w/w). Finally, fluorescently labelled non-modified (C), tuftsin-coated empty (CT) and tuftsin-oligomycin-loaded (CTO) nanoparticles were synthesized by adding Cy5 amine (Lumiprobe Corporation, Hunt Valley, MD) in DMSO to the aqueous dispersion of nanoparticles (equivalent to 0.16% of carboxylic groups in nanoparticles) in the presence of EDC and the mixture was incubated for 4 h in the dark. Unbound dye was removed by dialysis and fluorescence emission spectra of labelled nanoparticles were characterized using a FluoroMax-4 spectrofluorometer (HORIBA Scientific). Oligomycin release from CTO nanoparticles was determined using a PBS dialysis method with a 3.5 kDa membrane cutoff. The kinetics of oligomycin release was determined by HPLC and concentrations are expressed as a percentage of the total oligomycin available vs. time. Over 90% of oligomycin was released from CTO nanoparticles within a 24 h period at physiological pH (S2A Fig).

### Nanoparticle injection and IVIS imaging

Animals were placed in an induction chamber without restraint and anesthetized with 2.5% isoflurane. A total of 10 μg control (C or CT) or oligomycin (CTO) nanoparticles were administered by a single intra-articular injection (in 10 μL of PBS) at either day 3 or 7 post-infection. Nanoparticle injection and length of retention was evaluated in the same cohort of mice using an *I**n* *V**ivo* Imaging System (IVIS Spectrum; PerkinElmer, Waltham, MA) under isoflurane anesthesia, with excitation and emission wavelengths of 640 nm and 680 nm, respectively.

### Recovery of infected tissues for *S. aureus* enumeration

The titanium implant, femur, knee joint, and surrounding soft tissue were collected by first removing the skin, whereupon the tissue ventral to the patellar tendon was excised, weighed, and dissociated in 500 μL of PBS supplemented with a protease inhibitor cocktail tablet (Cat #88266 ThermoFisher, Waltham, MA). The knee joint and femur were processed separately using a hand-held homogenizer for 30 sec. The implant was removed and vortexed in 200 μL PBS to detach biofilm-associated bacteria. *S*. *aureus* titers were determined using TSA plates supplemented with 5% sheep blood (Cat #R01202 Remel, Lenexa, KS) and are expressed as Log_10_ (cfu/mL) for the titanium implant or Log_10_ (cfu/g) for tissues.

### Sample preparation and measurement of oligomycin content in tissues

Tissue samples harvested from sacrificed animals were weighed before homogenization, frozen, and stored at −80°C. Tissues were homogenized in PBS + protease inhibitor buffer using a TissueLyser (Qiagen). Oligomycin B (Cat #11343, Cayman Chemical) was used as an internal standard at a final concentration of 1 μg/mL. Homogenates were vortexed for 30 s, left for 5 min, then extracted using an excess (1 mL) of acetonitrile, vortexed for 3 min and placed on a shaker for an additional 15 min. All samples were then centrifuged at 10,000 x *g* for 10 min and the supernatants were transferred to a clean vial and evaporated to dryness, followed by reconstitution with 50 μL of 75% acetonitrile (LC-MS grade). Samples were run on an UPLC-MS system using an Acquity UPLC BEH C18 column (50 mm x 2.1 mm) and a mobile phase of 75% acetonitrile in water was applied under isocratic conditions at a flow rate of 0.5 mL/min. A UPLC-MS system equipped with a single quadrupole (Acquity QDA, Waters) coupled to a UPLC system (Acquity PDA, Waters) was used in multiple reaction monitoring (MRM) mode. The ESI-MS of Oligomycin A [C_45_H_73_O_11_Na]^+^ was calculated as 813.51 and was found as 813.79, whereas Oligomycin B [C_45_H_71_O_12_Na]^+^ was calculated as 827.49 and was found as 827.67. The retention times for Oligomycin A and Oligomycin B were 1.09 min and 0.77 min, respectively.

### JC-1 and 2-NBDG staining

Leukocytes recovered from animals with sterile or *S*. *aureus* infected orthopedic implants were stained with the bi-potential dye JC-1 (5,5',6,6'-Tetrachloro-1,1',3,3'-tetraethylbenzimidazolocarbocyanine iodide; Cat #T3168 Invitrogen) or the fluorescent glucose analog 2-NBDG (2-(N-(7-Nitrobenz-2-oxa-1,3-diazol-4-yl)Amino)-2-Deoxyglucose; Cat #N13195 ThermoFisher) to evaluate OxPhos and glycolytic activity, respectively. JC-1 emits red fluorescence in mitochondria with large membrane potentials and green fluorescence in depolarized mitochondria. Therefore, cells exhibiting increased OxPhos activity have a larger green:red ratio. Cells were incubated with either 100 ng/mL JC-1 or 10 μM 2-NBDG for 30 min at 37°C. Pooled aliquots of cells from each mouse (n = 5) ± FCCP treatment were used as compensation controls. Cells were washed and resuspended in 250 μL FACS buffer supplemented with 2 μL Fc-block (TruStain FcX, Cat #101320 BioLegend, San Diego, CA) to minimize nonspecific antibody binding. Finally, cells were stained with Live/Dead Fixable Blue Dead Cell Stain (Cat #L23105 Invitrogen, Eugene, OR), CD45-APC (Cat #103112; RRID:AB_312977), Ly6G-PacBlue (Cat #127612; RRID:AB_2251161), F4/80-PE-Cy7 (Cat #123114; RRID:AB_893478), and CD11b-AF700 (Cat #101222; RRID:AB_493705) (all from BioLegend), or Ly6C-PerCP-Cy5.5 (Cat #560525, BD Biosciences), to identify monocytes (CD11b^high^Ly6G^-^ Ly6C^+^F4/80^-^) as previously described [15]. OxPhos activity (JC-1) was calculated as the ratio of green:red monocytes and glycolysis was calculated as the percentage of 2-NBDG^+^ monocytes.

### Flow cytometry

To quantify biofilm-associated leukocytes in the soft tissue surrounding the infected knee, homogenates were filtered (35 μm; BD Falcon, BD Biosciences) and RBCs eliminated using RBC Lysis Buffer (Cat #420301, BioLegend). Cells were washed and incubated with TruStain FcX (Cat #101320, BioLegend) and stained with Live/Dead Fixable Blue Dead Cell Stain (Cat #L23105, Invitrogen), CD45-PacBlue (Cat #103126; RRID:AB_493535), Ly6G-PE (Cat #127608; RRID:AB_1186099), F4/80-PE-Cy7 (Cat #123114; RRID:AB_893478), and CD11b-FITC (Cat #101206; RRID:AB_312789) (all from BioLegend), and Ly6C-PerCP-Cy5.5 (Cat #560525, BD Pharmingen). ROS production was assessed using a CellROX Green kit (Cat #C10492, ThermoFisher) according to the manufacturer’s instructions. For individual samples, 10,000–100,000 events were analyzed using BD FACSDiva software (RRID:SCR_001456) with cell populations expressed as the percentage of total viable CD45^+^ leukocytes as previously described [15].

### Recovery of MDSCs and monocytes for gene expression analysis

Monocytes (CD11b^high^Ly6G^-^Ly6C^+^F4/80^-^) from the soft tissue surrounding the infected knee were purified by sorting on a FACSAria (BD Biosciences; RRID:SCR_016695) using the antibody panel described above, whereupon RNA was immediately isolated using an RNeasy Plus Micro Kit (Cat #74034 Qiagen, Hilden, Germany). cDNA was synthesized using the iScript cDNA Synthesis Kit (Cat #1708891 Bio-Rad, Hercules, CA) and qPCR was performed using TaqMan primer/probe mixes (ThermoFisher) for the following genes: arginase-1 (Arg-1; Cat #Mm00475988_m1), IL-10 (Cat #Mm00439616_m1), triggering receptor expressed on myeloid cells 2 (Trem2; Cat #Mm00451744_m1), hypoxia-inducible factor-alpha (HIF-1α; Cat #Mm00468869_m1), inducible nitric oxide synthase (iNOS; Cat #Mm00440485_m1), TNF-α (Cat #Mm00443258_m1), Trem1 (Cat #Mm00451738_m1), and glyceraldehyde-3-phosphate dehydrogenase (GAPDH; Cat #Mm99999915_g1). Gene expression levels of monocytes from CTO treated mice were normalized to GAPDH and are presented as the fold-induction (2^-ΔΔCt^) value relative to monocytes isolated from CT treated animals. In separate experiments, monocyte gene expression was evaluated by NanoString, using the nCounter PanCancer Mouse Immune Profiling kit (Cat #XT-CSO-MIP1-12; NanoString, Seattle, WA). Results are expressed as the fold-change in monocytes recovered from CTO vs. CT treated mice.

### Metabolomic analysis

Monocytes (CD11b^high^Ly6G^-^Ly6C^+^F4/80^-^) and MDSCs (CD11b^high^Ly6G^+^Ly6C^+^F4/80^-^) were recovered from animals receiving CT or CTO nanoparticles by FACS as described above and polar metabolites were extracted as previously reported [83]. Cell sorting by positive selection was not expected to elicit dramatic metabolic changes based on the rapid processing period and the fact that cells were maintained on ice to reduce their metabolic rate. In addition, the positive selection protocol was identical for monocytes and MDSCs recovered from CT and CTO treated animals, which would control for any potential effects of positive selection when making comparisons. Briefly, immediately after collection, monocytes and MDSCs were lysed using an 80% methanol/water mixture pre-chilled to -80°C. Cellular debris was removed by centrifugation at 13,000 rpm for 5 min at 4°C, whereupon supernatants containing metabolite extracts were collected and dried at room temperature using a speed vacuum concentrator (Eppendorf, Hamburg, Germany). Samples were resuspended in a 50% methanol/water mixture and analyzed using LC-MS/MS. A single reaction monitoring (SRM) LC-MS/MS method was used with positive/negative ion polarity switching on a Xevo TQ-S mass spectrometer as described previously [84]. The metabolite peak areas were calculated using MassLynx 4.1 (Waters Inc.) and normalized to the respective cell counts of each sample. The resultant peak areas were subjected to relative quantification analyses using MetaboAnalyst 3.0 (RRID:SCR_015539; www.metaboanalyst.ca). Fold-change values of metabolites were expressed as CTO relative to CT treated mice. Individual values exhibited a normal distribution and a Student’s *t*-test was used to determine significance. Principal component analysis, heat map, and pathway impact analysis were also performed using MetaboAnalyst 3.0 software.

A similar protocol described above was used to quantify intracellular metabolites from *S*. *aureus* planktonic and biofilm cultures treated with oligomycin. Planktonic cultures were grown for 2 h in RPMI-1640 supplemented with 10% dialyzed FBS (Cat #SH3007901, ThermoFisher) ± 10 μg/ml oligomycin, whereupon bacteria were pelleted by centrifugation. Biofilms were grown for 4 days before 10 μg/ml oligomycin was added for three additional days and bacteria were collected at day 7 by vigorous pipetting. For both planktonic and biofilm samples, bacteria were lysed using a bead beater (OMNI Bead Ruptor 24; Omni International) and intracellular extracts were processed and analyzed by LC/MS-MS as described above.

### Gentamicin protection assay

Mouse bone marrow-derived MΦs and thioglycollate-elicited peritoneal PMNs, or human monocyte-derived MΦs were cultured as described above. Mouse and human MΦs were seeded in a 96-well plate at 5x10^4^ cells/well and incubated overnight, while PMNs were isolated immediately before the experiment. Cells were pre-treated with free oligomycin (1 or 10 μg/mL) CT, or CTO nanoparticles (10 μg each) for 2 h before incubation with *S*. *aureus* at a MOI of 10:1 for 45 min at 37°C, 5% CO_2_. MΦs and PMNs were washed and resuspended in medium containing 100 μg/mL gentamicin (Cat #G1264, Sigma-Aldrich) and incubated for another 30 min to kill residual extracellular bacteria, whereupon cells were washed and medium containing 1 μg/mL gentamicin was added. Intracellular bacterial loads were determined at 0, 2, 4 and 6 h by lysing cells with sterile water and plating on blood agar.

### Statistics

Significant differences between experimental groups were determined by a one-way ANOVA with Bonferroni’s multiple comparisons. The statistical details for all experiments can be found in the accompanying figure legends along with the “n” and precision measures. Sample sizes were determined based on prior publications using groups that were sufficient to achieve a desired power of 0.85 and an alpha value of 0.05. All analyses were performed using GraphPad Prism version 6.04 (San Diego, CA; RRID:SCR_002798) and a *p*-value of < 0.05 was considered statistically significant.

## Supporting information

S1 Fig Oligomycin enhances leukocyte bactericidal activity. Mouse bone marrow-derived MФs and thioglycollate-elicited peritoneal neutrophils, or human monocyte-derived MФs were treated with various concentrations of free oligomycin or empty (CT) or oligomycin-containing (CTO) nanoparticles to evaluate the effects on *S*. *aureus* killing by gentamicin protection assays. Results are from one experiment (n = 8 biological replicates) and are presented as the mean ± SD. (*, *p* < 0.0001 for 0 vs. 1 μg/ml oligomycin; #, *p* < 0.0001 for 0 vs. 10 μg/ml oligomycin; †, *p* < 0.0001 for CT vs. CTO nanoparticles; Student’s *t*-test).
(TIF)

S2 Fig Kinetics of oligomycin release from tuftsin-modified nanoparticles and longevity of nanoparticle retention at the site of *S*. *aureus* biofilm infection. (**A**) Oligomycin release from Cy5-tuftsin-oligomycin (CTO) nanoparticles was determined by HPLC and concentrations are expressed as a percentage of the total oligomycin available vs. time (in hours; h). (**B**) C57BL/6NCrl mice received a single intra-articular injection of Cy5-labeled nanoparticles at day 3 post-infection, whereupon the same animal was imaged over a two-week period to demonstrate the extent of nanoparticle retention and distribution. Results are representative of 5 individual mice.
(TIF)

S3 Fig Kinetics of acute *S*. *aureus* orthopedic infection and identification of phagocytic leukocyte populations. (**A**) C57BL/6NCrl mice (n = 4–5) were inoculated with 1,000 cfu *S*. *aureus*, whereupon bacterial burden was determined at days 1–3 post-infection and normalized to tissue weight where indicated. (**B**) Mice (n = 10) were infected with 1,000 cfu of a *S*. *aureus* USA300 LAC-dsRed reporter strain and dsRed^+^ MDSCs, PMNs, and monocytes were assessed at day 3 post-infection as a measure of phagocytosis. Results are expressed as the percentage of dsRed^+^ cells relative to each leukocyte population (mean ± SD).
(TIF)

S4 Fig Oligomycin does not alter neutrophil activation. Mouse thioglycollate-elicited peritoneal neutrophils were treated with various concentrations of oligomycin for 2 h, whereupon TNF-α and IL-10 as well as arginase activity were determined by cytometric bead array, ELISA, and an enzymatic assay, respectively. Results are combined from two independent experiments (n = 4–16 biological replicates) and are presented as the mean ± SD.
(TIF)

S5 Fig Oligomycin-containing nanoparticles do not significantly affect *S*. *aureus* biofilm burden until 7 days post-infection. C57BL/6NCrl mice received a single intra-articular injection of Cy5 (C), Cy5/Tuftsin (CT), or Cy5/Tuftsin/Oligomycin (CTO) nanoparticles at day 7 post-infection, whereupon animals were sacrificed 3 or 7 days following nanoparticle treatment. Bacterial burden was quantified from the (**A**) surrounding soft tissue, (**B**) knee, (**C**) femur, and (**D**) implant. Results are from one experiment (n = 5 mice/group/time point). (*, *p* < 0.05; **, *p* < 0.01; ***, *p* < 0.001; One-way ANOVA).
(TIF)

S6 Fig Oligomycin-containing nanoparticles polarize monocytes towards a pro-inflammatory phenotype *in vivo*. C57BL/6NCrl mice received a single intra-articular injection of Cy5/Tuftsin (CT) or Cy5/Tuftsin/Oligomycin (CTO) nanoparticles (10 μg) at day 7 post-infection. Mice were sacrificed 3 days following nanoparticle injection and monocytes (CD11b^high^Ly6G^-^Ly6C^+^F4/80^-^) were sorted from pooled samples (n = 5 mice/treatment group) by FACS, whereupon RNA was immediately isolated for NanoString analysis. Gene expression levels in monocytes recovered from CTO-treated animals are presented as the fold-change relative to monocytes isolated from mice receiving CT (control) nanoparticles. Data is presented as the mean ± SD (n = 2 sets of monocytes) from two independent experiments.
(TIF)

S7 Fig Nanoparticle-mediated delivery of oligomycin to monocytes at 3 days post-infection reduces biofilm burden. (**A**) C57BL/6NCrl mice received a single intra-articular injection of Cy5 (C), Cy5/Tuftsin (CT), or Cy5/Tuftsin/Oligomycin (CTO) nanoparticles at day 3 post-infection, and were analyzed out to 28 days following nanoparticle treatment. Bacterial burden was quantified in the (**B**) surrounding soft tissue, (**C**) knee, (**D**) femur, and (**E**) implant, where the dotted line represents the limit of detection (LOD). Infiltrating leukocytes were analyzed by flow cytometry and (**F**) MDSCs, (**G**) PMNs, (**H**) monocytes, and (**I**) macrophages are reported as the percentage of live CD45^+^ leukocytes (mean ± SD). Results are combined from three independent experiments (n = 15 mice/group/time point). (*, *p* < 0.05; **, *p* < 0.01; ***, *p* < 0.001; ****, *p* < 0.0001; One-way ANOVA).
(TIF)

S8 Fig Oligomycin lacks *S*. *aureus* antibacterial activity *in vivo*. (**A**) C57BL/6NCrl mice received a single intra-articular injection of free oligomycin only (Oligo; 100 ng), empty nanoparticles (CT), empty nanoparticles (CT) + free oligomycin (100 ng; not loaded), or oligomycin loaded nanoparticles (CTO) at day 7 post-infection and bacterial burden was assessed in the surrounding soft tissue, knee, femur, and implant at day 3 (5 mice/group) or day 7 (10 mice/group) after treatment, where the dotted lines represent the limit of detection (LOD; **, *p* < 0.01; ****, *p* < 0.0001; One-way ANOVA). (**B**) C57BL/6NCrl mice received one intra-articular injection of oligomycin at 7 day post-infection (100 ng), two sequential doses at days 7 & 8 post-infection (50 ng/day), or vehicle (PBS) and were sacrificed at day 14 post-infection. Bacterial burden was quantified from the surrounding soft tissue, knee, femur, and implant. Results are from one experiment (n = 5 mice/group/time point). (*, *p* < 0.05; One-way ANOVA).
(TIF)

S9 Fig Oligomycin lacks *S*. *aureus* antibacterial activity *in vitro*. *S*. *aureus* was exposed to various concentrations of oligomycin during (**A**) the initiation of biofilm culture (time 0) and throughout the 4 day maturation period, (**B**) treatment of mature biofilms for 4 days, or (**C**) planktonic growth beginning at time 0. Biofilm cultures were replenished daily with fresh medium containing oligomycin. Results are presented as (**A** and **B**) Log_10_ colony forming units (CFU) per well (mean ± SD) or (**C**) OD_600_ from one experiment (n = 5 and n = 10 biological replicates for biofilm and planktonic cultures, respectively).
(TIF)

S10 Fig Oligomycin has minimal effects on *S*. *aureus* metabolism during biofilm or planktonic growth. (**A**, **B**, and **E**) Mature *S*. *aureus* biofilms (day 4 of growth) were treated with 10 μg/ml oligomycin for 3 days, whereupon bacteria were collected. (**C**, **D**, and **F**) Oligomycin (10 μg/ml) was added to a planktonic *S*. *aureus* culture at time 0 and bacteria were collected 2 h later. In both cases, the intracellular metabolome was quantified by LC/MS-MS and compared to bacteria without oligomycin treatment. (**A and C**) Principle component analysis (PCA) plots for biofilm and planktonic growth were generated using an algorithm in MetaboAnalyst with mean intensities and pareto scaling distribution. Ellipses represent a 95% confidence interval of the normal distribution for each cluster. (**B and D**) The heat maps depict the top metabolite differences in biofilm or planktonic cultures treated with oligomycin versus vehicle, respectively. The color key indicates log_2_-fold changes of normalized mean peak intensities for metabolites in biofilms or planktonic cultures ± oligomycin. (**E and F**) Graphical representation of the metabolites significantly affected by oligomycin treatment during biofilm or planktonic growth, respectively (*p* < 0.05).
(TIF)

S11 Fig *S*. *aureus* glycolytic metabolism is not affected by oligomycin during either biofilm or planktonic growth. (**A**) Mature biofilms (day 4 of growth) were treated with 10 μg/ml oligomycin for 3 days, whereupon bacteria were collected at day 7. (**B**) Oligomycin (10 μg/ml) was added to a planktonic *S*. *aureus* culture at time 0 and bacteria were collected 2 h later to evaluate glycolytic intermediates by LC/MS-MS and were compared to bacteria without oligomycin treatment. ND, not detected.
(TIF)

S12 Fig Free oligomycin is not detectable in joint-associated tissue following oligomycin nanoparticle injection *in vivo*. (**A**) UPLC-MS analysis of oligomycin A + B standards, each at a concentration of 0.5 μg/mL. (a) Selective ion recording (SIR) chromatogram of Oligomycin B; (b) SIR chromatogram of Oligomycin A; (c) UV (225 nm) chromatogram of Oligomycin B and Oligomycin A with retention times of 0.77 min and 1.095 min, respectively. Scale in SIR chromatogram is 0–2100; 0–0.025 in UV chromatogram. (**B**) UPLC-MS analysis of tissue samples spiked with 5 ng of Oligomycin A and 50 ng of Oligomycin B (internal standard) at a final concentration of 0.1 μg/mL and 1 μg/mL, respectively. (a) SIR chromatogram of Oligomycin B; (b) SIR chromatogram of Oligomycin A (scale in the graph insert is 0–250); (c) UV chromatogram of Oligomycin B and Oligomycin A. (**C**) C57BL/6NCrl mice received a single intra-articular injection of Cy5/Tuftsin/Oligomycin (CTO) nanoparticles (10 μg) at day 7 post-infection (n = 5 mice/treatment group) and were sacrificed 3 days following nanoparticle injection to quantify free oligomycin in joint-associated tissues using the methodology described in **A** and **B**.
(TIF)

S13 Fig Monocyte metabolic reprogramming with oligomycin-containing nanoparticles cooperates with antibiotics to clear established *S*. *aureus* biofilm infection. C57BL/6NCrl mice received a single intra-articular injection of Cy5/Tuftsin (CT) or Cy5/Tuftsin/Oligomycin (CTO) nanoparticles (10 μg) at day 7 post-infection (n = 5/group). Seven days later, animals received daily i.p. injections of antibiotics (Abx; 25 mg/kg/day rifampin and 5 mg/kg/day daptomycin) or vehicle (Veh) for one week, whereupon mice were sacrificed at day 28 post-infection. Bacterial burden was quantified from the (**A**) surrounding soft tissue, (**B**) knee, (**C**) femur, and (**D**) implant, where the dotted line represents the limit of detection (LOD). (*, *p* < 0.05; **, *p* < 0.01; ***, *p* < 0.001; ****, *p* < 0.0001; One-way ANOVA).
(TIF)
