# Supplementary figures and images for: Monocyte metabolic reprogramming promotes pro-inflammatory activity and Staphylococcus aureus biofilm clearance
Source: PLoS Pathog. 2020 Mar 6;16(3):e1008354. doi: 10.1371/journal.ppat.1008354 (PMC7080272; doi:10.1371/journal.ppat.1008354)

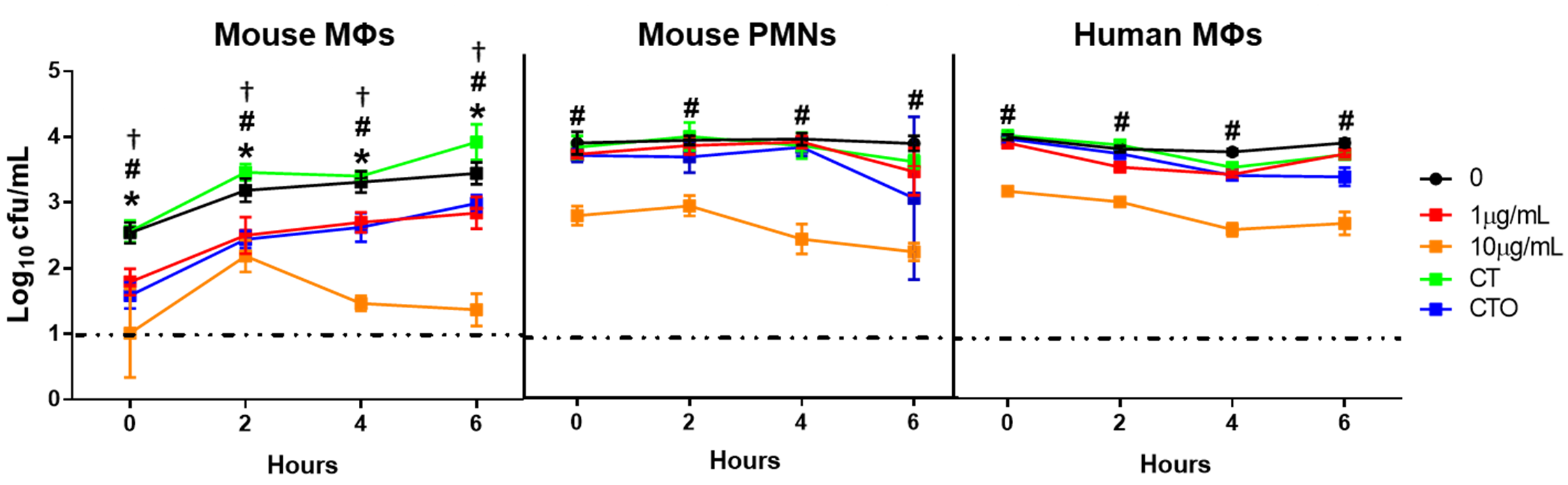

Supplement: S1 Fig — Mouse bone marrow-derived MФs and thioglycollate-elicited peritoneal neutrophils, or human monocyte-derived MФs were treated with various concentrations of free oligomycin or empty (CT) or oligomycin-containing (CTO) nanoparticles to evaluate the effects on S. aureus killing by gentamicin protection assays. Results are from one experiment (n = 8 biological replicates) and are presented as the mean ± SD. (*, p < 0.0001 for 0 vs. 1 μg/ml oligomycin; #, p < 0.0001 for 0 vs. 10 μg/ml oligomycin; †, p < 0.0001 for CT vs. CTO nanoparticles; Student’s t-test). (TIF) [file ppat.1008354.s001.tif]

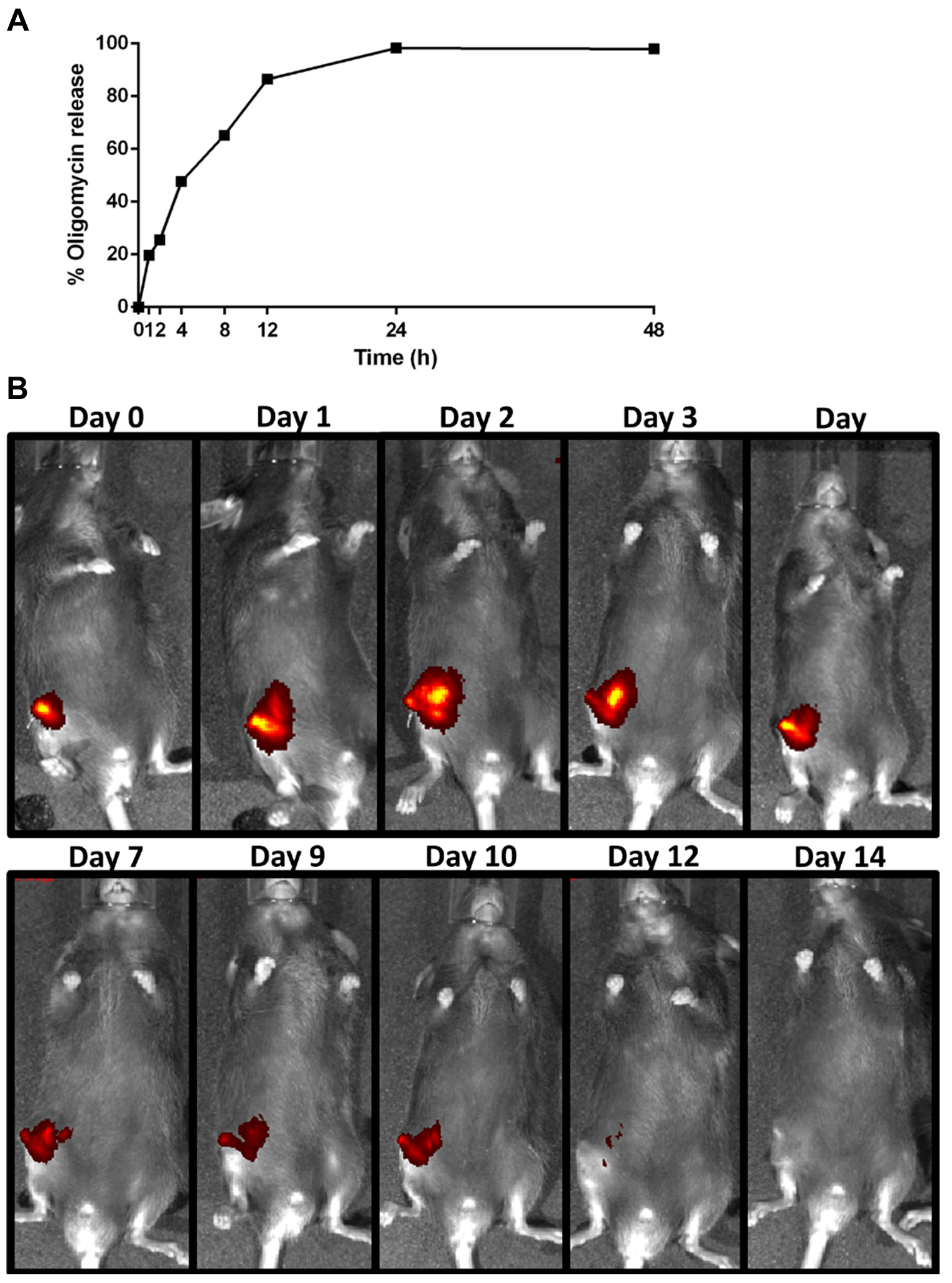

Supplement: S2 Fig — (A) Oligomycin release from Cy5-tuftsin-oligomycin (CTO) nanoparticles was determined by HPLC and concentrations are expressed as a percentage of the total oligomycin available vs. time (in hours; h). (B) C57BL/6NCrl mice received a single intra-articular injection of Cy5-labeled nanoparticles at day 3 post-infection, whereupon the same animal was imaged over a two-week period to demonstrate the extent of nanoparticle retention and distribution. Results are representative of 5 individual mice. (TIF) [file ppat.1008354.s002.tif]

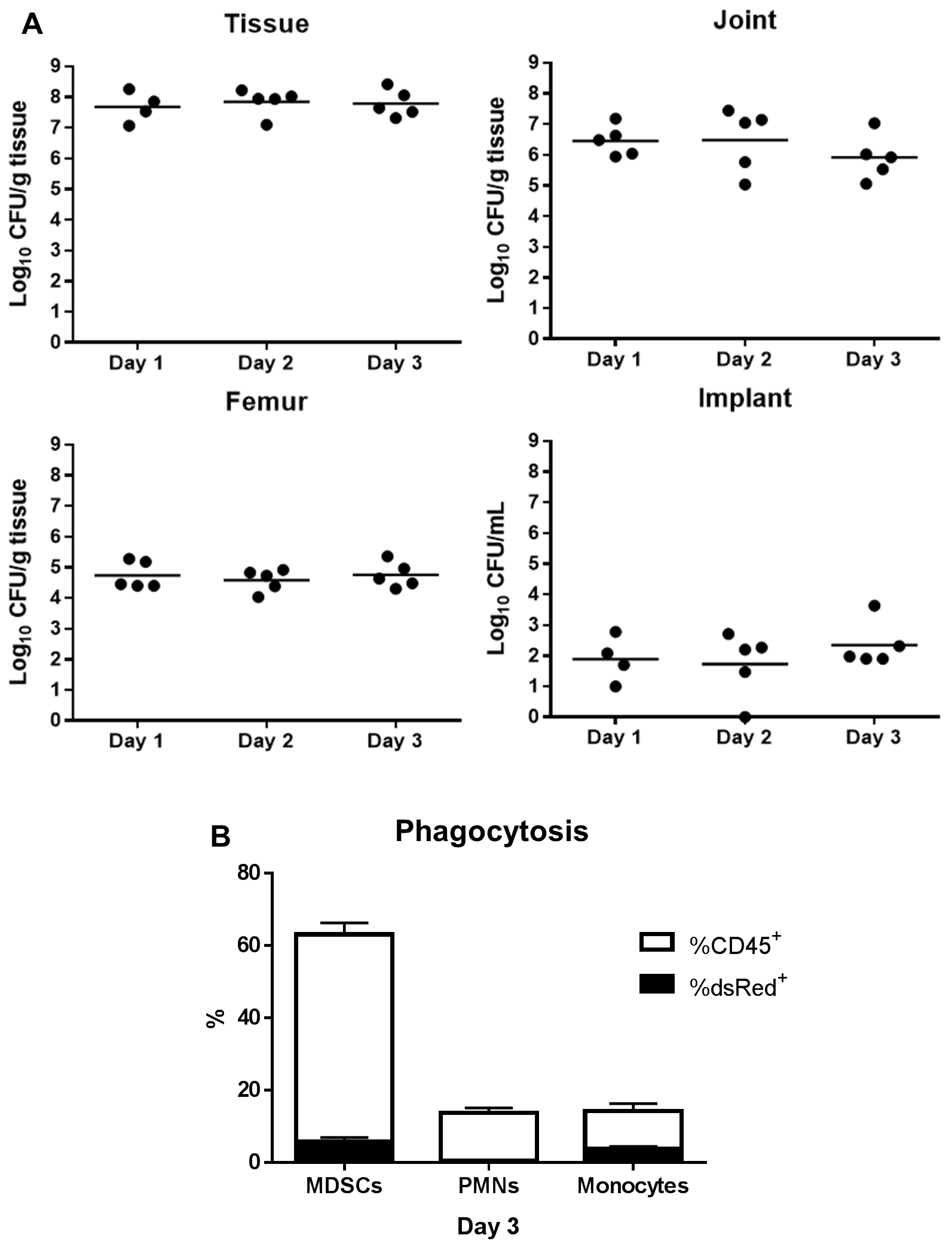

Supplement: S3 Fig — (A) C57BL/6NCrl mice (n = 4–5) were inoculated with 1,000 cfu S. aureus, whereupon bacterial burden was determined at days 1–3 post-infection and normalized to tissue weight where indicated. (B) Mice (n = 10) were infected with 1,000 cfu of a S. aureus USA300 LAC-dsRed reporter strain and dsRed+ MDSCs, PMNs, and monocytes were assessed at day 3 post-infection as a measure of phagocytosis. Results are expressed as the percentage of dsRed+ cells relative to each leukocyte population (mean ± SD). (TIF) [file ppat.1008354.s003.tif]

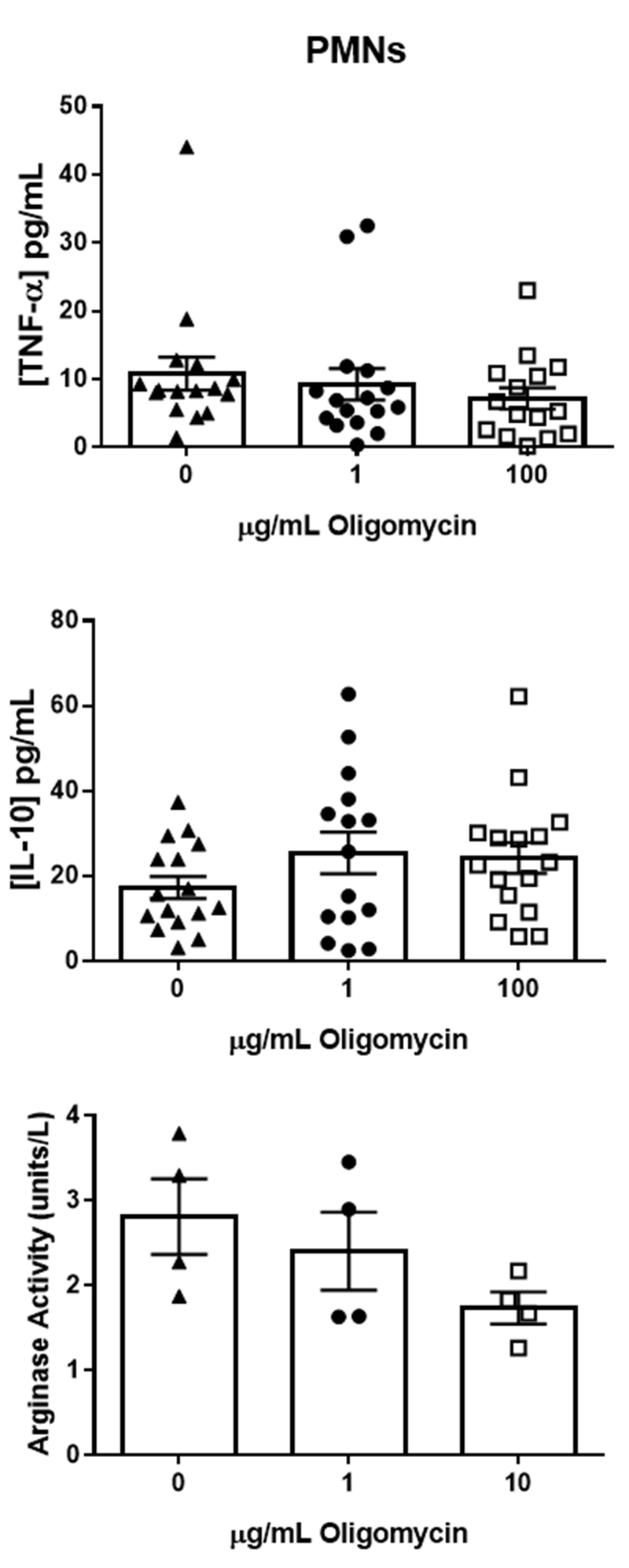

Supplement: S4 Fig — Mouse thioglycollate-elicited peritoneal neutrophils were treated with various concentrations of oligomycin for 2 h, whereupon TNF-α and IL-10 as well as arginase activity were determined by cytometric bead array, ELISA, and an enzymatic assay, respectively. Results are combined from two independent experiments (n = 4–16 biological replicates) and are presented as the mean ± SD. (TIF) [file ppat.1008354.s004.tif]

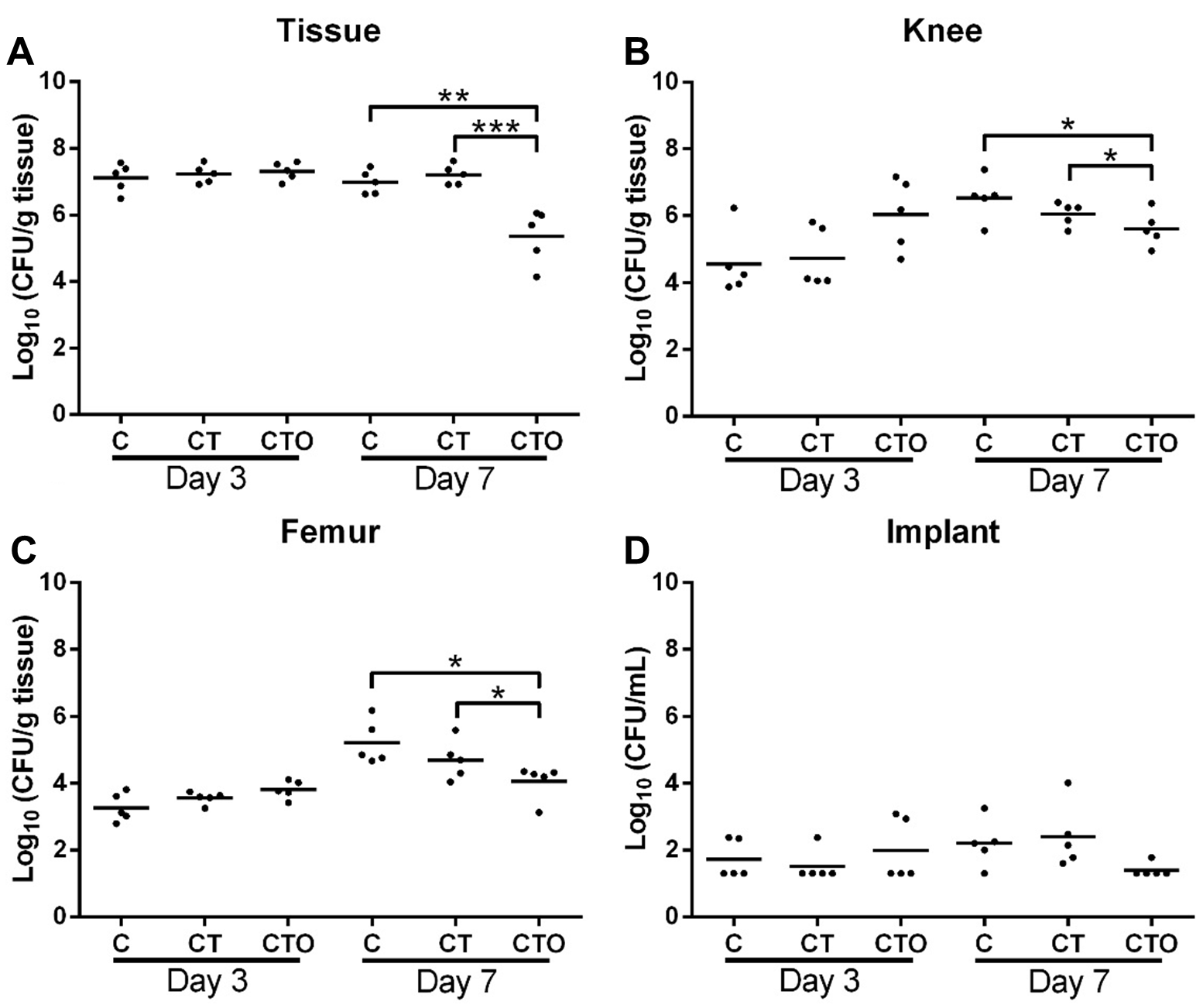

Supplement: S5 Fig — C57BL/6NCrl mice received a single intra-articular injection of Cy5 (C), Cy5/Tuftsin (CT), or Cy5/Tuftsin/Oligomycin (CTO) nanoparticles at day 7 post-infection, whereupon animals were sacrificed 3 or 7 days following nanoparticle treatment. Bacterial burden was quantified from the (A) surrounding soft tissue, (B) knee, (C) femur, and (D) implant. Results are from one experiment (n = 5 mice/group/time point). (*, p < 0.05; **, p < 0.01; ***, p < 0.001; One-way ANOVA). (TIF) [file ppat.1008354.s005.tif]

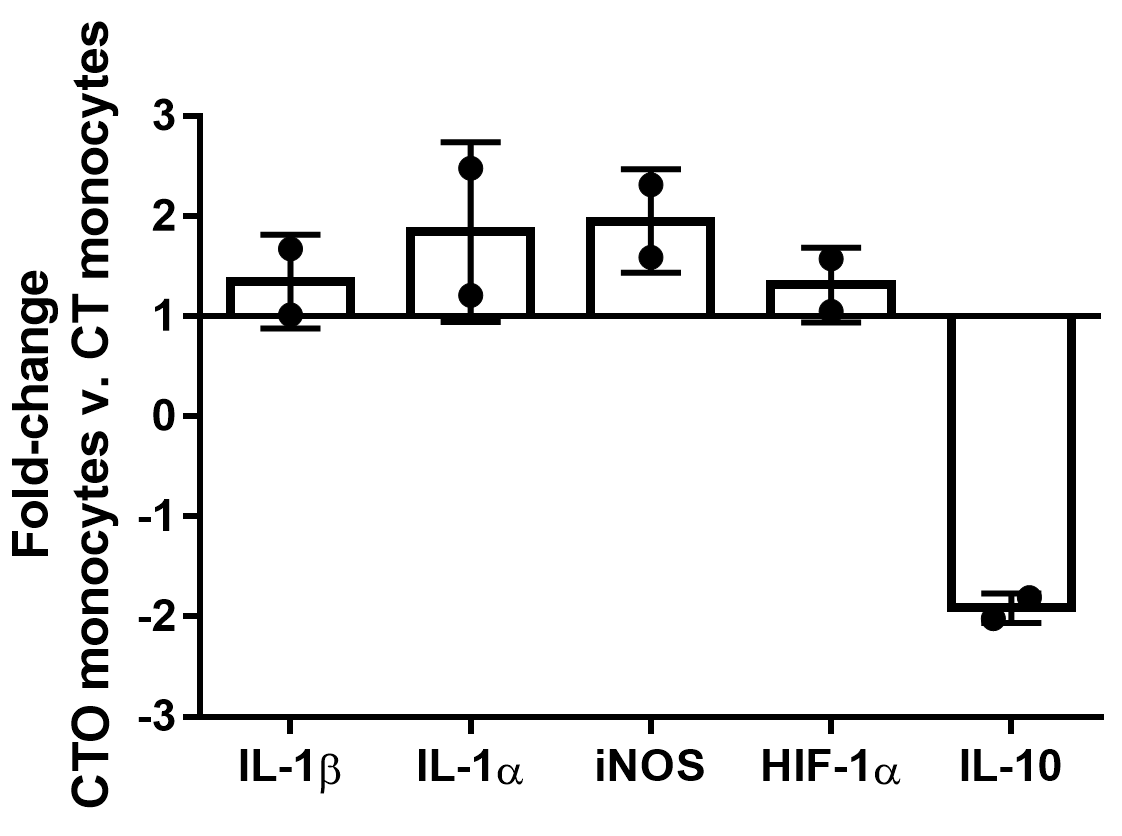

Supplement: S6 Fig — C57BL/6NCrl mice received a single intra-articular injection of Cy5/Tuftsin (CT) or Cy5/Tuftsin/Oligomycin (CTO) nanoparticles (10 μg) at day 7 post-infection. Mice were sacrificed 3 days following nanoparticle injection and monocytes (CD11bhighLy6G-Ly6C+F4/80-) were sorted from pooled samples (n = 5 mice/treatment group) by FACS, whereupon RNA was immediately isolated for NanoString analysis. Gene expression levels in monocytes recovered from CTO-treated animals are presented as the fold-change relative to monocytes isolated from mice receiving CT (control) nanoparticles. Data is presented as the mean ± SD (n = 2 sets of monocytes) from two independent experiments. (TIF) [file ppat.1008354.s006.tif]

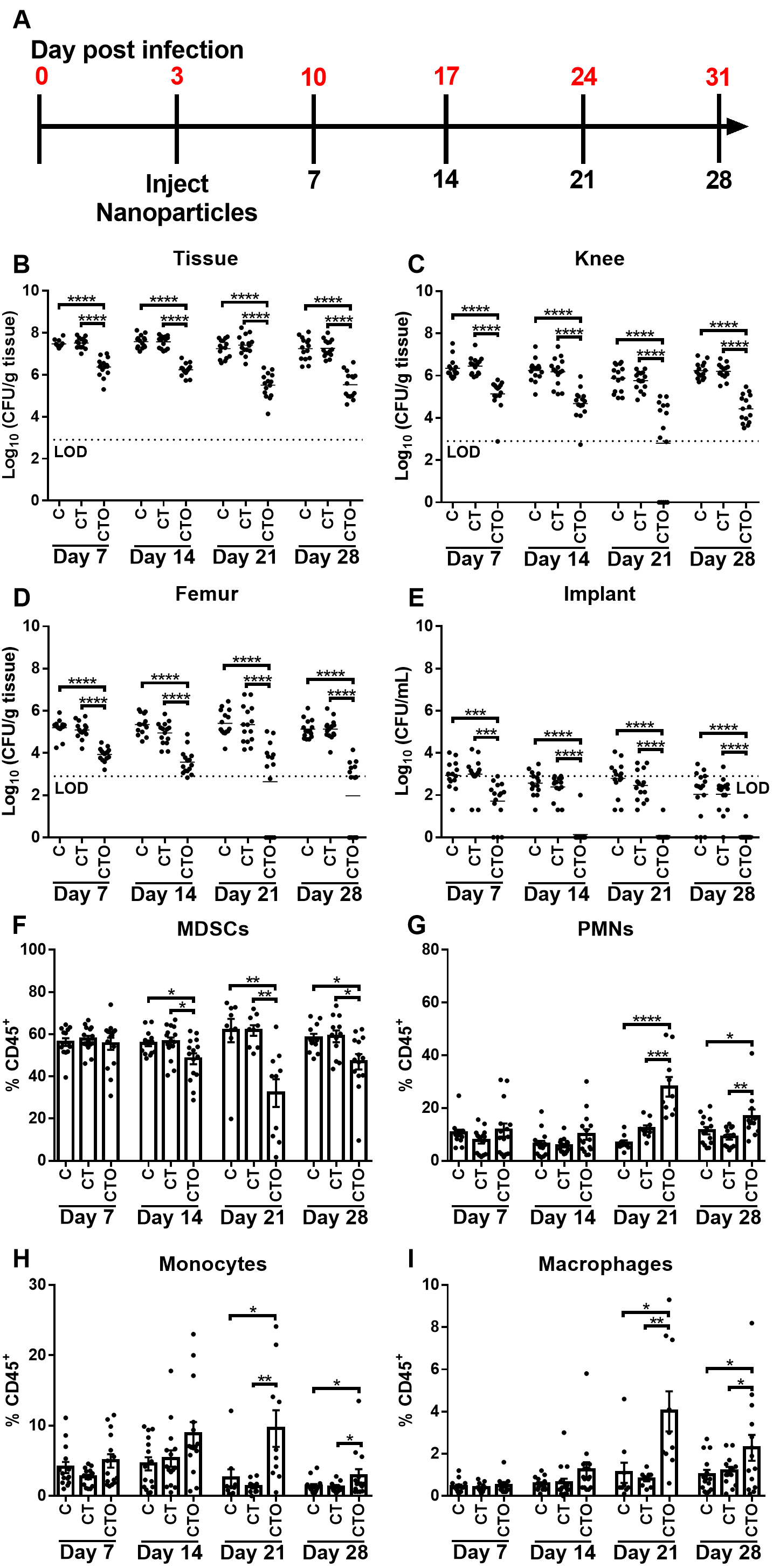

Supplement: S7 Fig — (A) C57BL/6NCrl mice received a single intra-articular injection of Cy5 (C), Cy5/Tuftsin (CT), or Cy5/Tuftsin/Oligomycin (CTO) nanoparticles at day 3 post-infection, and were analyzed out to 28 days following nanoparticle treatment. Bacterial burden was quantified in the (B) surrounding soft tissue, (C) knee, (D) femur, and (E) implant, where the dotted line represents the limit of detection (LOD). Infiltrating leukocytes were analyzed by flow cytometry and (F) MDSCs, (G) PMNs, (H) monocytes, and (I) macrophages are reported as the percentage of live CD45+ leukocytes (mean ± SD). Results are combined from three independent experiments (n = 15 mice/group/time point). (*, p < 0.05; **, p < 0.01; ***, p < 0.001; ****, p < 0.0001; One-way ANOVA). (TIF) [file ppat.1008354.s007.tif]

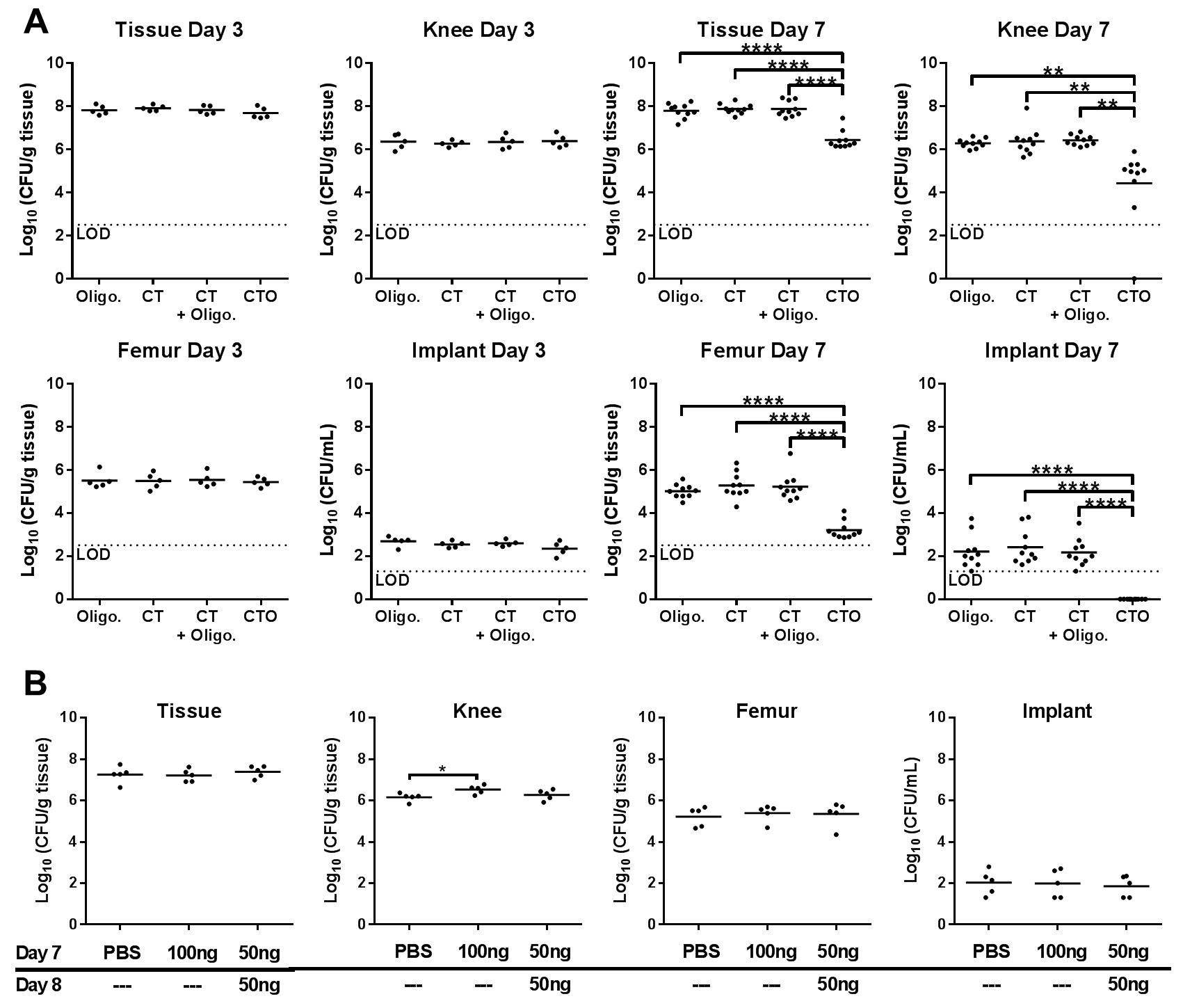

Supplement: S8 Fig — (A) C57BL/6NCrl mice received a single intra-articular injection of free oligomycin only (Oligo; 100 ng), empty nanoparticles (CT), empty nanoparticles (CT) + free oligomycin (100 ng; not loaded), or oligomycin loaded nanoparticles (CTO) at day 7 post-infection and bacterial burden was assessed in the surrounding soft tissue, knee, femur, and implant at day 3 (5 mice/group) or day 7 (10 mice/group) after treatment, where the dotted lines represent the limit of detection (LOD; **, p < 0.01; ****, p < 0.0001; One-way ANOVA). (B) C57BL/6NCrl mice received one intra-articular injection of oligomycin at 7 day post-infection (100 ng), two sequential doses at days 7 & 8 post-infection (50 ng/day), or vehicle (PBS) and were sacrificed at day 14 post-infection. Bacterial burden was quantified from the surrounding soft tissue, knee, femur, and implant. Results are from one experiment (n = 5 mice/group/time point). (*, p < 0.05; One-way ANOVA). (TIF) [file ppat.1008354.s008.tif]

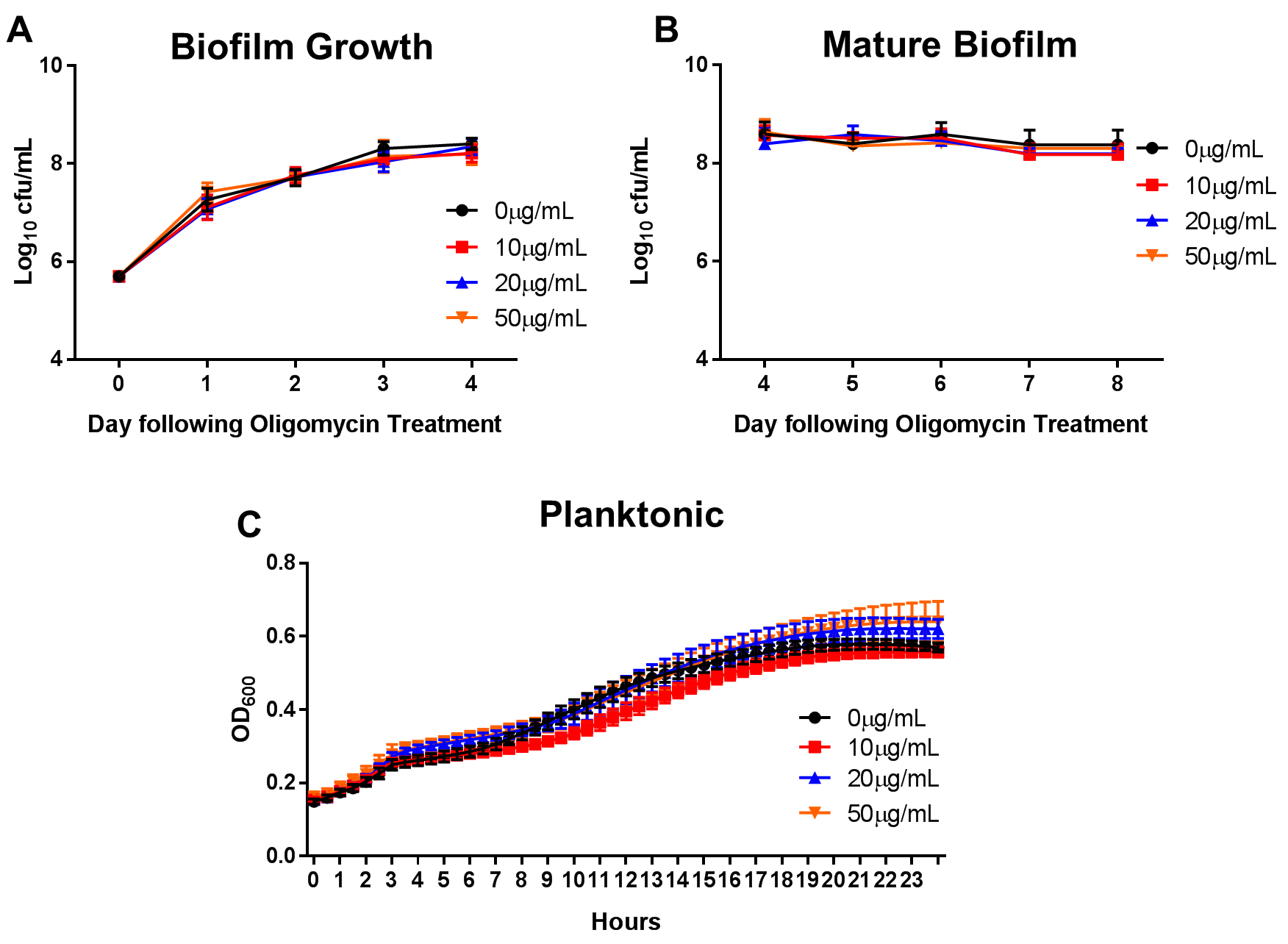

Supplement: S9 Fig — S. aureus was exposed to various concentrations of oligomycin during (A) the initiation of biofilm culture (time 0) and throughout the 4 day maturation period, (B) treatment of mature biofilms for 4 days, or (C) planktonic growth beginning at time 0. Biofilm cultures were replenished daily with fresh medium containing oligomycin. Results are presented as (A and B) Log10 colony forming units (CFU) per well (mean ± SD) or (C) OD600 from one experiment (n = 5 and n = 10 biological replicates for biofilm and planktonic cultures, respectively). (TIF) [file ppat.1008354.s009.tif]

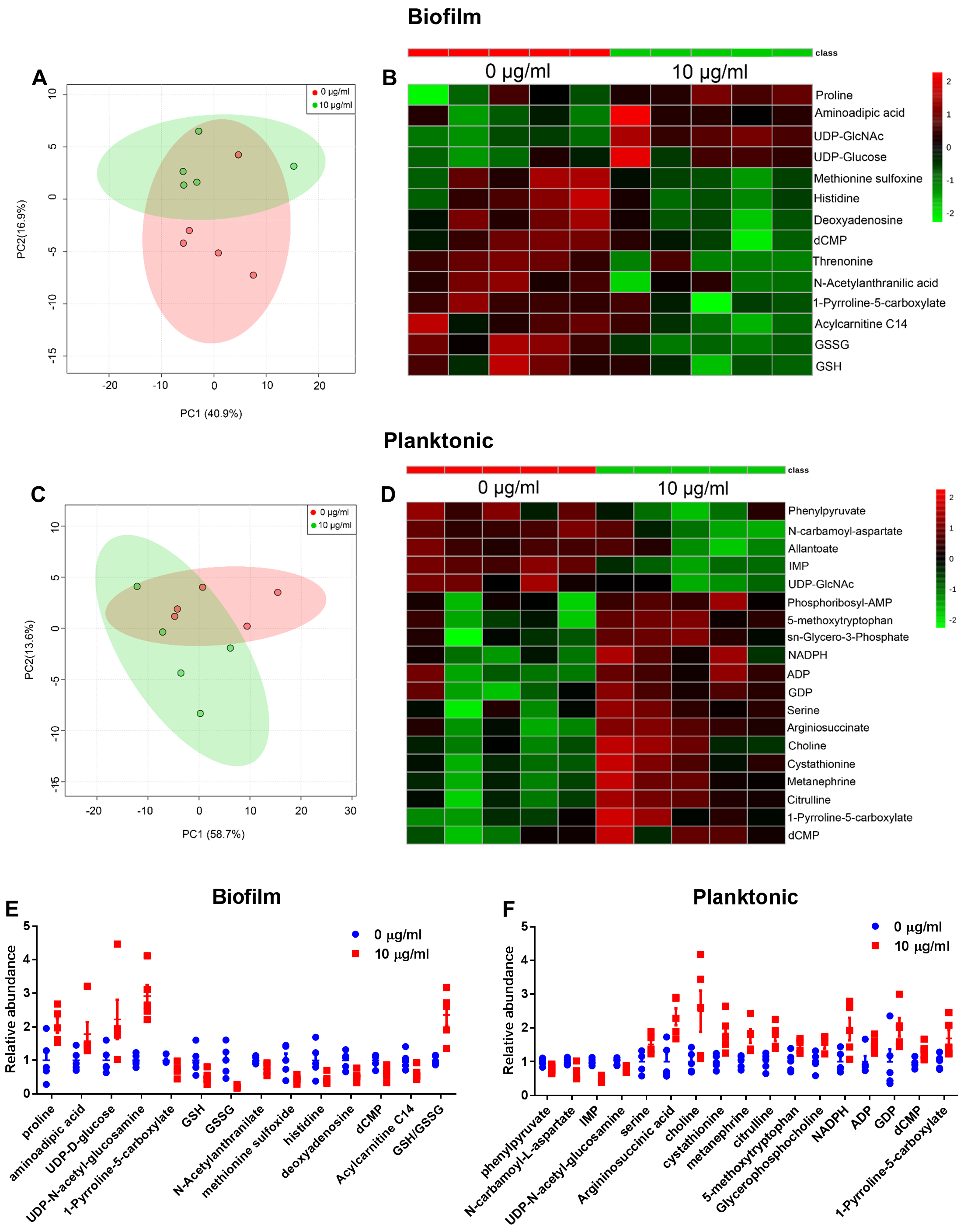

Supplement: S10 Fig — (A, B, and E) Mature S. aureus biofilms (day 4 of growth) were treated with 10 μg/ml oligomycin for 3 days, whereupon bacteria were collected. (C, D, and F) Oligomycin (10 μg/ml) was added to a planktonic S. aureus culture at time 0 and bacteria were collected 2 h later. In both cases, the intracellular metabolome was quantified by LC/MS-MS and compared to bacteria without oligomycin treatment. (A and C) Principle component analysis (PCA) plots for biofilm and planktonic growth were generated using an algorithm in MetaboAnalyst with mean intensities and pareto scaling distribution. Ellipses represent a 95% confidence interval of the normal distribution for each cluster. (B and D) The heat maps depict the top metabolite differences in biofilm or planktonic cultures treated with oligomycin versus vehicle, respectively. The color key indicates log2-fold changes of normalized mean peak intensities for metabolites in biofilms or planktonic cultures ± oligomycin. (E and F) Graphical representation of the metabolites significantly affected by oligomycin treatment during biofilm or planktonic growth, respectively (p < 0.05). (TIF) [file ppat.1008354.s010.tif]

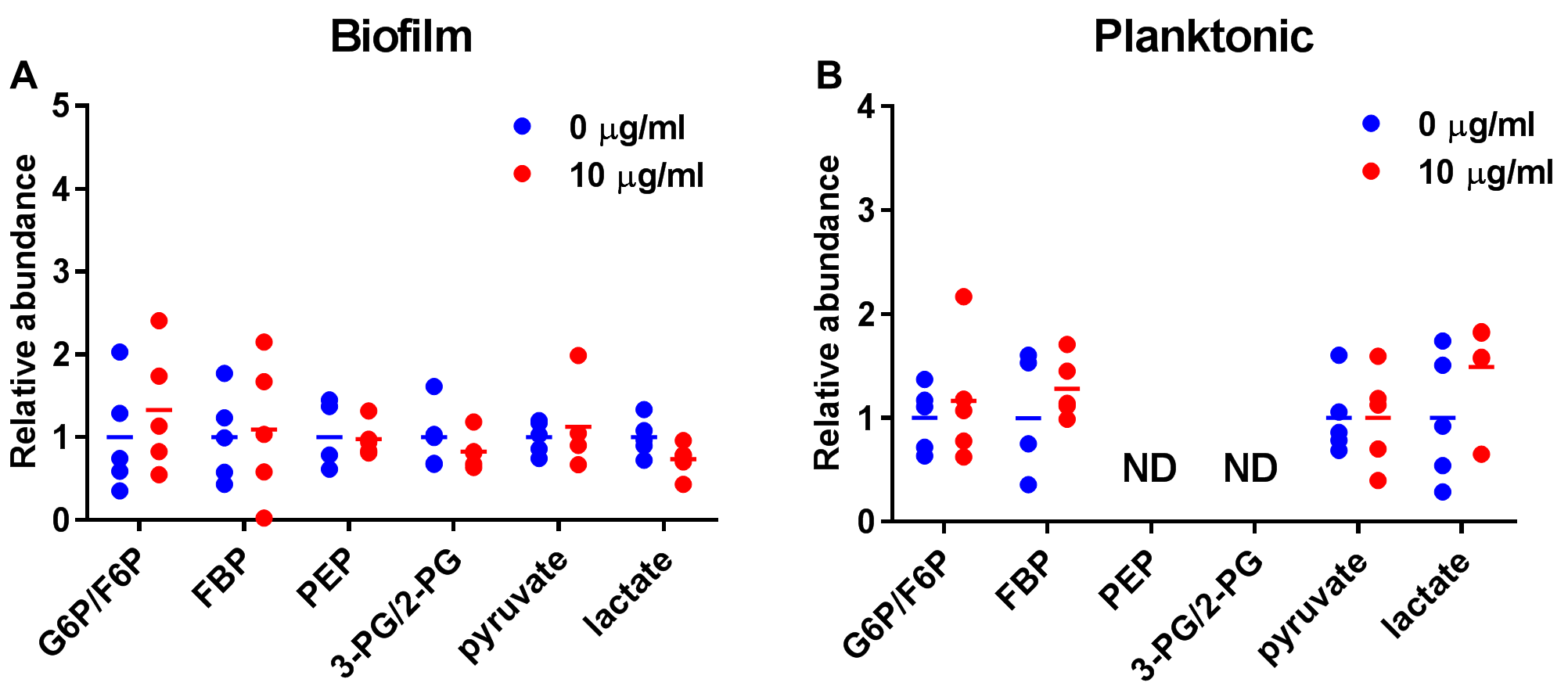

Supplement: S11 Fig — (A) Mature biofilms (day 4 of growth) were treated with 10 μg/ml oligomycin for 3 days, whereupon bacteria were collected at day 7. (B) Oligomycin (10 μg/ml) was added to a planktonic S. aureus culture at time 0 and bacteria were collected 2 h later to evaluate glycolytic intermediates by LC/MS-MS and were compared to bacteria without oligomycin treatment. ND, not detected. (TIF) [file ppat.1008354.s011.tif]

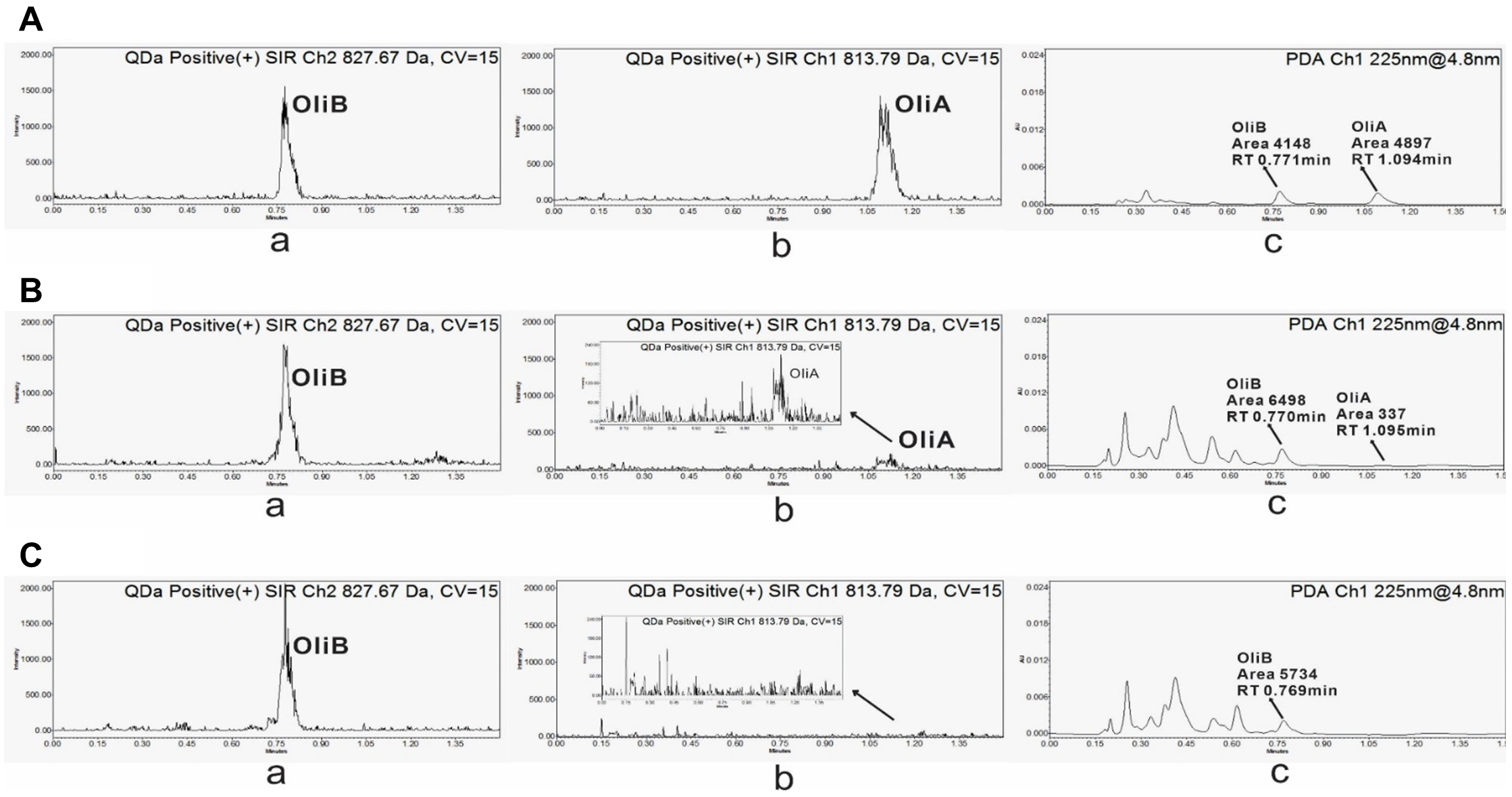

Supplement: S12 Fig — (A) UPLC-MS analysis of oligomycin A + B standards, each at a concentration of 0.5 μg/mL. (a) Selective ion recording (SIR) chromatogram of Oligomycin B; (b) SIR chromatogram of Oligomycin A; (c) UV (225 nm) chromatogram of Oligomycin B and Oligomycin A with retention times of 0.77 min and 1.095 min, respectively. Scale in SIR chromatogram is 0–2100; 0–0.025 in UV chromatogram. (B) UPLC-MS analysis of tissue samples spiked with 5 ng of Oligomycin A and 50 ng of Oligomycin B (internal standard) at a final concentration of 0.1 μg/mL and 1 μg/mL, respectively. (a) SIR chromatogram of Oligomycin B; (b) SIR chromatogram of Oligomycin A (scale in the graph insert is 0–250); (c) UV chromatogram of Oligomycin B and Oligomycin A. (C) C57BL/6NCrl mice received a single intra-articular injection of Cy5/Tuftsin/Oligomycin (CTO) nanoparticles (10 μg) at day 7 post-infection (n = 5 mice/treatment group) and were sacrificed 3 days following nanoparticle injection to quantify free oligomycin in joint-associated tissues using the methodology described in A and B. (TIF) [file ppat.1008354.s012.tif]

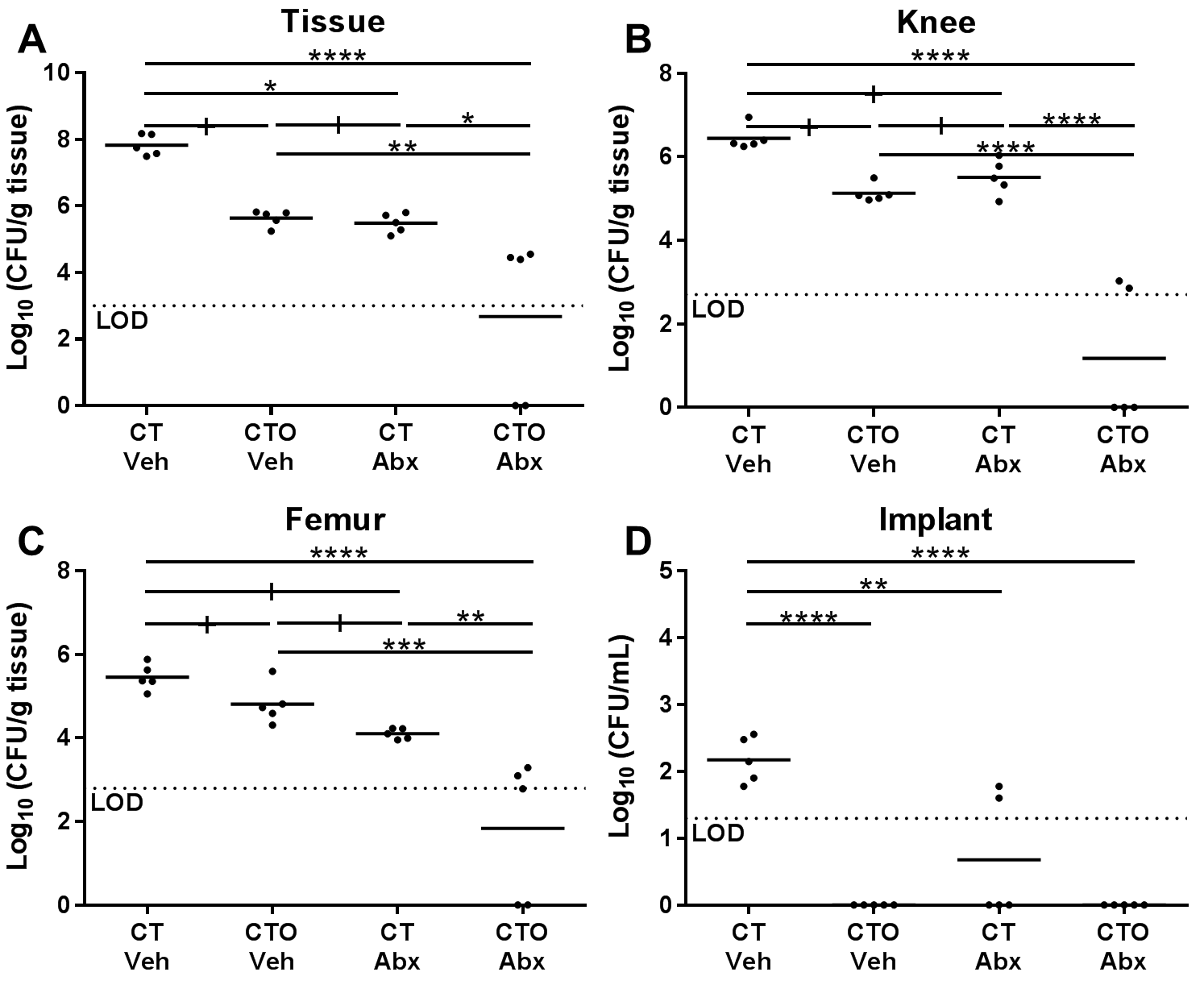

Supplement: S13 Fig — C57BL/6NCrl mice received a single intra-articular injection of Cy5/Tuftsin (CT) or Cy5/Tuftsin/Oligomycin (CTO) nanoparticles (10 μg) at day 7 post-infection (n = 5/group). Seven days later, animals received daily i.p. injections of antibiotics (Abx; 25 mg/kg/day rifampin and 5 mg/kg/day daptomycin) or vehicle (Veh) for one week, whereupon mice were sacrificed at day 28 post-infection. Bacterial burden was quantified from the (A) surrounding soft tissue, (B) knee, (C) femur, and (D) implant, where the dotted line represents the limit of detection (LOD). (*, p < 0.05; **, p < 0.01; ***, p < 0.001; ****, p < 0.0001; One-way ANOVA). (TIF) [file ppat.1008354.s013.tif]
